# Structural insight into host plasma membrane association and assembly of HIV-1 matrix protein

**DOI:** 10.1038/s41598-021-95236-8

**Published:** 2021-08-04

**Authors:** Halilibrahim Ciftci, Hiroshi Tateishi, Kotaro Koiwai, Ryoko Koga, Kensaku Anraku, Kazuaki Monde, Çağdaş Dağ, Ebru Destan, Busra Yuksel, Esra Ayan, Gunseli Yildirim, Merve Yigin, F. Betul Ertem, Alaleh Shafiei, Omur Guven, Sabri O. Besler, Raymond G. Sierra, Chun Hong Yoon, Zhen Su, Mengling Liang, Burcin Acar, Turkan Haliloglu, Masami Otsuka, Fumiaki Yumoto, Mikako Fujita, Toshiya Senda, Hasan DeMirci

**Affiliations:** 1 Medicinal and Biological Chemistry Science Farm Joint Research Laboratory, Faculty of Life Sciences, Kumamoto University, Kumamoto, 862-0973 Japan; 2 Department of Drug Discovery, Science Farm Ltd, Kumamoto, 862-0976 Japan; 3 Stanford PULSE Institute, SLAC National Accelerator Laboratory, Menlo Park, CA 94025 USA; 4 Structural Biology Research Center, Institute of Materials Structure Science, KEK/High Energy Accelerator Research Organization, Tsukuba, Ibaraki 305-0801 Japan; 5 Department of Medical Technology, Kumamoto Health Science University, Kumamoto, 861-5598 Japan; 6 Department of Microbiology, Faculty of Life Sciences, Kumamoto University, Kumamoto, 860-8556 Japan; 7 Department of Molecular Biology and Genetics, Koc University, 34450 Istanbul, Turkey; 8 Linac Coherent Light Source, SLAC National Accelerator Laboratory, Menlo Park, CA USA; 9 Department of Applied Physics, Stanford University, Stanford, CA USA; 10 Department of Chemical Engineering, Bogazici University, 34342 Istanbul, Turkey; 11 Polymer Research Center, Bogazici University, 34342 Istanbul, Turkey; 12 School of High Energy Accelerator Science, SOKENDAI University, Tsukuba, Ibaraki 305-0801 Japan; 13 Faculty of Pure and Applied Sciences, University of Tsukuba, Ibaraki, 305-8571 Japan; 14 Koc University Isbank Center for Infectious Diseases (KUISCID), 34450 Istanbul, Turkey

**Keywords:** X-ray crystallography, Structural biology

## Abstract

Oligomerization of Pr55^Gag^ is a critical step of the late stage of the HIV life cycle. It has been known that the binding of IP6, an abundant endogenous cyclitol molecule at the MA domain, has been linked to the oligomerization of Pr55^Gag^. However, the exact binding site of IP6 on MA remains unknown and the structural details of this interaction are missing. Here, we present three high-resolution crystal structures of the MA domain in complex with IP6 molecules to reveal its binding mode. Additionally, extensive Differential Scanning Fluorimetry analysis combined with cryo- and ambient-temperature X-ray crystallography and GNM-based transfer entropy calculations identify the key residues that participate in IP6 binding. Our data provide novel insights about the multilayered HIV-1 virion assembly process that involves the interplay of IP6 with PIP2, a phosphoinositide essential for the binding of Pr55^Gag^ to membrane. IP6 and PIP2 have neighboring alternate binding sites within the same highly basic region (residues 18–33). This indicates that IP6 and PIP2 bindings are not mutually exclusive and may play a key role in coordinating virion particles’ membrane localization. Based on our three different IP6-MA complex crystal structures, we propose a new model that involves IP6 coordination of the oligomerization of outer MA and inner CA domain’s 2D layers during assembly and budding.

## Introduction

Viruses are one of the most harmful pathogens to humanity. With their limited number of genes, they can tactfully utilize the host-cell machinery for replication via interaction between viral proteins and host-cell targets^1^. Human immunodeficiency virus type 1 (HIV-1), which carries only nine genes, replicates in T4-helper cells^2^, thus causing acquired immunodeficiency syndrome (AIDS) by utilizing components of the host cell in each step of its life cycle. G*roup-specific antigen* (*gag*) encodes polyprotein precursor (Pr55^Gag^) which initiates the assembly of viral proteins and viral genomic RNA for the budding of virions. Pr55^Gag^ contains the p6 domain that hijacks requisite host proteins (Supplementary Fig. 1). Virus release has (1) Gag membrane binding (2) Gag-assembly and (3) the budding process, encompassing multimerization of viral proteins and their assembly at the host’s cellular membrane. This process involves the recruitment of key host proteins to allow for viral budding and facilitates the recruitment of other essential components necessary for viral infection such as lipids and nucleic acids^3,4^. Therefore, Pr55^Gag^ is an important molecule which can be targeted for the treatment of HIV.

At the beginning of the HIV-1 replication cycle, Pr55^Gag^ is synthesized from a viral genomic RNA transcript inside the host cell. Pr55^Gag^ consists of four domains: the matrix domain (MA), capsid domain (CA), nucleocapsid domain (NC) and p6 domain respectively. In addition, Pr55^Gag^ has two small spacer peptides SP1 between CA and NC, and SP2 between NC and p6 which influence viral release and infectivity (Supplementary Fig. 2). Pr55^*Gag*^ is directed to the plasma membrane through the MA domain and is incorporated into viral envelope (Env) glycoproteins to create virion particles^5,6^. Moreover, MA is crucial for the proper conformation of HIV-1 Gag protein. The N-terminal domain of MA conjugated with the C-terminal domain of NC forms a horseshoe-like conformation of Gag protein^7^. Virion maturation is the final step of late stage infection and is initiated by the cleavage characterized by separation of Gag domains through the action of a specific viral protease^3,4^.

The MA domain of Pr55^*Gag*^ contains five α-helices: four of which form an N-terminal globular head while the fifth α-helix forms a C-terminal helix that is connected to the CA domain^8^. The MA domain has two signals for Gag integration into the host plasma membrane. One is the "highly basic region" (HBR), which includes residues 18–33. As the other signal, the assembly of Pr55^Gag^ at the plasma membrane requires myristoylation of the MA domain at its N-terminus^9^. Moreover, 1-D-*myo*-phosphatidylinositol 4,5-bisphosphate [PI(4,5)P2] (PIP2) is a native host membrane phospholipid recognized via the MA domain of Pr55^Gag^ during the localization of membrane proteins^10^. The HBR within the N-terminus of MA has an essential role, leading hotspot activity by providing necessary interaction between MA and PIP2^11,12^. In addition, MA has different observed oligomeric states: trimer, hexamer and higher-order oligomers on the host plasma membrane^13^. As any structural ablation of the higher-order state will lead to viral release inhibition, it makes the N-terminus amino acid sequence critical^14^.

Detailed high-resolution structural studies of MA-inositol complexes are still not available and impedes the further understanding of the assembly of Pr55^Gag^ at the plasma membrane. Unlike previous studies^15–17^, high-resolution studies in this direction will offer invaluable opportunities for the molecular design of lead high-efficiency compounds that may inhibit the completion of the HIV cycle by blocking the Pr55^Gag^-PIP2 interaction^18^. Studies such as the solution NMR structure of MA-PIP2 complex with shorter lipophilic chains revealed that a basic pocket of the MA domain accommodates acidic inositol phosphate moieties by electrostatic interactions^19^. Previous studies suggested that the other inositol molecules such as inositol hexakisphosphate (phytic acid, IP6) in addition to PIP2 are essential for the canonical HIV-1 particle assembly process^20^. IP6 is an abundant endogenous molecule in organisms and has critical roles in biological activities such as the IP signaling cascade. Although it was thought previously to be present only in plants, it has been discovered that IP6 is also very common within the animal kingdom^21^. As an abundant host metabolite, IP6 is hijacked for the benefit of HIV-1 through interaction with amino acid residues within the SP1 region of Pr55^Gag^ ^7,22^. Furthermore, IP6 is involved in the assembly of Pr55^Gag^ by binding to both MA and NC domains, each of which are responsible for the encapsidation of the viral genome into virions and facilitate assembly and encapsidation^23^. The binding of IP6 to the CA domain increases HIV-1 capsid stability and RNA accumulation, leading to viral production. Therefore, the endogenous concentration of IP6 is critical for HIV-1 assembly^22^. In the presence of IP6, HIV-1 Gag performs conformational transition into virus-like particles (VLPs). During the assembly process of Pr55^Gag^, Inositol phosphates (IPs) are generated to correct the radius of curvature. This leads to alteration of VLPs to achieve mature conformation^7,23^.

It is essential to determine the high-resolution structures of the MA-IP6 complexes to obtain molecular insight into the dynamic interaction between MA and IP6 at the molecular level. Here we employed both synchrotron cryo X-ray crystallography and ambient-temperature Serial Femtosecond X-ray crystallography (SFX) at an X-ray Free Electron Laser (XFEL) to obtain MA-IP6 co-crystal structures. We report three crystal forms and resulting X-ray structures of MA in complex with IP6 molecule (MA_IP6). One of our crystal structures reveals a novel form of a hexameric state of MA in the presence of IP6. Our data demonstrate that the binding site of MA-IP6 is different from the PIP2-binding site and through this interaction, IP6 shows significant importance in the oligomerization of MA. Our results are also supported by extensive net Transfer Entropy (nTE) analysis of monomeric, trimeric, hexameric and dodecamer structures of MA protein. Accordingly, the IP6 binding site directly affects trimerization, PIP2 binding, envelope interaction, and myristoylation sites in a monomeric state. Moreover, IP6, PIP2, myristoyl group, and the C-terminal of the domain interact with trimerization sites mutually in both trimeric and hexameric states. In conclusion, our data suggest that IP6 binding of MA regulates Gag protein localization and higher-order oligomerization on the host membrane even in the monomeric or hexameric state of MA. These findings may provide a better understanding of the HIV-1 replication mechanism besides a novel insight into the interaction between MA and IP6.

## Materials and methods

### Reagents

IP6 was purchased from Merck (Merck, Darmstadt, Germany). The reagents used for the crystallographic studies were ultrapure molecular biology grade and purchased from Sigma-Aldrich, USA.

### Cloning and overexpression of the HIV-1 Gag MA domain

A protocol for bacterial expression and purification of the MA domain is previously described^26^. A gene encoding MA fusion with Tobacco Etch Virus (TEV) protease cleavage site at the N-terminus site was subcloned between *Kpn*I (5’-terminus) and *Hind*III (3’-terminus) restriction enzyme sites in the pRSF-1b vector (Merck, Darmstadt, Germany) to express His-TEV protease site-MA protein (pRSF-1b_MA) (10His-tagged MA).

### Protein expression and purification

10His-tagged MA gene was expressed in *Escherichia coli* (*E. coli*) strain BL21 (DE3) (Merck, Darmstadt, Germany). 10His-tagged MA protein was initially purified with nickel-NTA affinity column chromatography followed by TEV cleavage, denaturation, refolding, and size exclusion column chromatography. *E. coli* BL21 (DE3) transformed with pRSF-1b_MA was cultured in LB broth supplemented by 50 mg/mL kanamycin at 37 °C. When the culture reached OD_600_ of approximately 0.6, target protein expression was induced by 0.1 mM Isopropyl β-D-1-thiogalactopyranoside (IPTG) at 16 °C overnight. Harvested cells were resuspended in a lysis buffer containing 20 mM Tris–HCl (pH 8.0), 500 mM NaCl, 30 mM Imidazole, 5 mM β-mercaptoethanol and lysed by sonication (ULTRA5 HOMOGENIZER VP-305, TAITEC, Output 7, Duty 50%, 2 min). The lysate was separated into pellet and supernatant by centrifugation at 15,000 g for 30 min at 4 °C, and the supernatant was applied to a nickel-NTA affinity column containing a total of 4 ml bed volume of nickel-NTA superflow resin (Qiagen, Venlo, Netherlands). After washing with a washing buffer containing 20 mM Tris–HCl, pH 8.0, 1 M NaCl, 1 M (NH_4_)_2_SO_4_, 30 mM Imidazole, 5 mM β-mercaptoethanol, the target protein was eluted with 15 ml of an elution buffer that contains 20 mM Tris–HCl (pH 8.0), 500 mM NaCl, 1 M (NH_4_)_2_SO_4_, 5 mM β-mercaptoethanol, 300 mM Imidazole. The eluent was concentrated to 2 ml, and 10His tag was removed by 0.1 mg/ml 6His-tagged TEV protease while dialyzed overnight in dialysis buffer containing 20 mM Tris–HCl (pH 8.0), 500 mM NaCl, 5% glycerol, 1 mM dithiothreitol (DTT). The mixture was diluted to 50 ml, and the His-tagged TEV protease, cleaved 10His tag and uncleaved 10His-tagged MA were separated by the reverse Ni–NTA affinity chromatography. The cleaved un-tagged product was collected at the flow-through fraction of the affinity chromatography. 2 ml of fraction was mixed with 10 ml of denaturation buffer containing 20 mM Tris–HCl (pH 8.0), 1 M NaCl, 1 M (NH_4_)_2_SO_4_, 1 mM DTT, 6 M urea and the protein was denatured overnight during dialysis in the denaturing buffer. After concentrating to a final volume of 1 ml, polypeptides were purified with size exclusion chromatography (SEC) column Superdex 200 10/300 increase (GE Healthcare, Little Chalfont, United Kingdom) which equilibrated with the SEC buffer containing 20 mM Tris–HCl (pH 8.0), 150 mM NaCl, 1 mM DTT using Akta FPLC system (GE Healthcare) at 0.2 mL/min. Peak fractions containing refolded MA were collected and concentrated to a final concentration of 10 mg/ml for crystallization.

### Crystallization for cryo synchrotron studies

200 mM Phosphatidylinositol-6-phosphate (IP6) was mixed with 10 mg/ml MA protein solution. Protein Crystallization System (PXS) was used for the initial crystallization screening of the MA-IP6 complex^26^^.^ In total, 384 conditions were examined using Crystal Screen 1 & 2 (Hampton Research), Index (Hampton Research), PEG-Ion (Hampton Research), and PEG-Ion2 (Hampton Research), and Wizard I & II (Molecular Dimensions). First crystallization condition contained 25% (w/v) PEG 3350 and 100 mM MES (pH 6.0) (MA_IP6_1), and the second one contained 5% (w/v) PEG 4000, 20% (v/v) 2-propanol and 100 mM MES (pH 6.5).

### Cryogenic data collection and processing

Crystals were harvested with the proper size of MicroLoops (MiTeGen, New York, United States of America), flash frozen by plunging into liquid nitrogen and transferred in Uni-pucks for automated data collection. Diffraction data were collected at the beamline BL-1A in the Photon Factory. The diffraction data sets were automatically processed and scaled using *XDS*^27^, *POINTLESS*^28^, and *AIMLESS*^29^. Crystallographic statistics are summarized in Supplementary Table 1.

### Crystal structure determination and refinement of cryogenic synchrotron structures

Phases for all three MA_IP6 structures’ data were determined by the molecular replacement method using the crystal structure of MA (PDB ID: 1HIW) as a search model with *MOLREP*^30^. Molecular models were initially refined with *REFMAC5*^31^ and further refined by *PHENIX.refine*^32^. Molecular models were manually built by *COOT*^33^. m*F*o—D*F*c omit-maps for ligands were calculated using *PHENIX* with a simulated annealing protocol. Interaction between MA and IP6 was analyzed by *PISA*^34^. All molecular graphics in this manuscript were prepared by the *PyMOL* Molecular Graphics System, Version 2.3.0 Schrödinger, LLC.

### Batch crystallization of the HIV-1 Gag MA domain for SFX

Purified HIV-1 Gag MA proteins were used in co-crystallization with IP6 at room temperature by the hanging-drop method using a crystallization buffer containing 20% (w/v) polyethylene glycol 3350 (PEG 3350) as a precipitant and 100 mM MES-NaOH (pH 6.5)^35^. Microcrystals were harvested in the same mother-liquor composition, pooled to a total volume of 3 ml and filtered through a 40-micron Millipore mesh filter. The concentration of crystal was around 10^10^–10^11^ per milliliter viewed by light microscopy.

### X-ray free electron laser data collection parameters

An average of 2.64 mJ was delivered in each 40-fs pulse containing approximately 10^12^ photons with 9.51 keV photon energy with 1 × 1 mm^2^ focus of X-rays. Single-pulse diffraction patterns from HIV-1 MA-IP6 microcrystals were recorded at 120 Hz on a CSPAD^36^ detector positioned at a distance of 217 mm from the interaction region.

### Sample delivery of MA-IP6 microcrystals into an XFEL and data collection

A crystalline slurry of MA-IP6 microcrystals kept at ambient-temperature flowing at 2 µl/min was injected into the interaction region inside the front vacuum chamber at the LCLS CXI instrument using the coMESH injector^37^. Large size crystals were present in the MA-IP6 sample slurry, prior to the experiment. The coMESH injector required filtered samples before injection through a 100-micron inner diameter capillary size to prevent clogging.

### Hit finding and indexing

SFX diffraction data collected from rod-shaped crystals at LCLS were processed using *Psocake*^38,39^, yielding a complete dataset. A diffraction pattern was deemed a hit if at least 15 peaks were found. A total of 126,434 diffraction patterns for MA-IP6 microcrystals were recorded as crystal hits. *CrystFEL*’s *indeximajig* program^40,41^ was used to index the crystal hits (Supplementary Fig. 2). Two rounds of indexing were performed on the dataset. Initial indexing results indicated that the space group was most likely triclinic *P*1 with a = 96.73 Å, b = 96.97 Å, c = 91.76 Å and *α* = 90.05°, *β* = 90°, *γ* = 120°. Given the target unit cell, the indexing results were accepted if the unit cell lengths and angles were within 5% and 1.5°, respectively. The final iteration yielded 56,861 (45%) indexed patterns for the MA-IP6 dataset.

### Differential scanning fluorimetry assay

A protocol for Differential Scanning Fluorimetry (DSF) was employed according to the previous procedures^42,43^. SYPRO® Orange (Thermo Fisher Scientific, Massachusetts, United States of America) solution (× 5000) was diluted by 50 times with a buffer containing 50 mM MOPS-NaOH (pH 7.0), 50 mM NaCl (100 × SYPRO® Orange solution). A buffer solution containing purified His10-tagged MA protein was exchanged to the 50 mM MOPS-NaOH, 50 mM NaCl buffer (pH 7.0) with ultrafiltration using Amicon® Ultra-15 Centrifugal Filter Unit (10 kDa MWCO) (Merck, Darmstadt, Germany). With the prepared 100 × SYPRO® Orange solution and the protein solution, 50 μl of a reaction mixture were prepared to be 5.4 µM MA-His10 protein, 5 × SYPRO® Orange in presence of or in absence of the indicated concentration of compounds to evaluate the contribution of IP6 to the thermostability of MA protein, or 32.4 µM IP6 for MA point-mutant analysis. The temperature of the reaction system was increased 0.5 °C per 30 s from 20 to 95 °C stepwise, and fluorescence intensity at each temperature was measured by Single-Color Real-Time PCR Detection System, MyiQ (Bio-Rad, California, United States of America), and analyzed with iQ5 (Bio-Rad).

### Gaussian network model (GNM)-based transfer entropy calculations

The Gaussian Network Model (GNM) is the most minimalist isotropic elastic network model of Cα atoms with harmonic interactions for the dynamics of proteins and their complexes^44,45^. GNM-based Transfer Entropy (TE) calculations^46,47^ reveal causal interrelations of residues in a given structural topology by considering a certain time delay τ between the fluctuations *∆R*_i_ (t) of residue i at time t and *∆R*_j_ (t + **τ**) of residue j at time t + **τ**. Using these fluctuations, TE(i,j) (**τ**) provides an estimate for the direction of information flow from residue i to residue j in time delay **τ**; i.e. TE(i,j) (**τ**) describes how much the present movement of residue i decreases the amount of uncertainty for the future movement of residue j within time interval **τ**. If TE(i,j)(**τ**) > TE(j,i)(**τ**), the dynamics of residue i affects the dynamics of residue j, indicating a causal directional interrelation from residue i to j in time delay **τ**.

The transfer entropy TE(i,j) (τ) from each residue pairs of i at time t and j at time t + **τ** was formulated 46 (Hacisuleyman and Erman, 2017) as:

1 $$TE(i,j)(\tau ) = S(\Delta R_{j} (t + \tau )\left| {\Delta R_{i} (t)} \right.) - S(\Delta R_{j} (t + \tau )\left| {\Delta R_{i} (t),} \right.\Delta R_{j} (t))$$

where the conditional entropies are:

2 $$(\Delta R_{i} (t + \tau )\left| {\Delta R_{i} (t)} \right. = - \left\langle {\ln p(\Delta R_{j} (0),\Delta R_{j} (\tau ))} \right\rangle + \left\langle {\ln p(\Delta R_{j} (0))} \right\rangle$$

3 $$S(\Delta R_{j} (t + \tau )\left| {\Delta R_{i} (t),} \right.\Delta R_{j} (t) = - \left\langle {\ln p(\Delta R_{j} (0),\Delta R_{j} (\tau ))} \right\rangle + \left\langle {\ln p(\Delta R_{i} (0),\Delta R_{j} (0),\Delta R_{j} (\tau ))} \right\rangle$$

Each term in Eqs. (2) and (3) could be obtained using GNM^46^, which mainly bases the calculations on the topology of the structure by computing the connectivity/Kirchoff matrix from cartesian coordinates. Time delay **τ** between fluctuations ∆*R*_i_ and ∆*R*_j_ is used as the four-fold of the defined time variable that maximizes mean transfer entropies over all residue pairs i and j.

GNM decomposes the motions into a spectrum of dynamic modes, from global/slow (low frequency) modes to local/fast (high frequency) modes. TE values in Eq. 1 could be calculated using all or a subgroup of dynamic modes^47^. As the global modes of motion are highly relevant for functional dynamics^48^, here we considered the slowest end of the dynamic mode spectrum with and without the slowest mode; average ten slowest and average two to ten slowest dynamic modes.

To emphasize the dynamically affecting and affected residues of each residue pair i and j, netTE (nTE) values is defined as

4 $$nTE_{ij} \left( \tau \right) = TE_{ij} \left( \tau \right) - TE_{ji} \left( \tau \right)$$

nTE is displayed in color (from blue to red as transfer entropies ascend) for each residue pair i and j on transfer entropy heat maps (Supplementary Fig. 8–12). For representative residues (highlighted in grey), their causal interrelation with the rest of residues is color-coded from highest positive (red) to lowest negative (blue) nTE values on all used topologies, given next to the nTE heat maps.

To identify affecting residues as entropy sources with the capacity to affect the others at the most, i.e. transfer information to the others, the cumulative net transfer entropy (cnTE) values of residue i over all j residues were also calculated as

5 $$cnTE\left( {i,\tau } \right) = \Sigma_{j} \left( {TE_{ij} \left( \tau \right) - TE_{ji} \left( \tau \right)} \right)$$

(cnTE) values of residue pairs are given as a line plot on the left of transfer entropy heatmaps (Supplementary Fig. 6–10). The maximally affected residues are defined as entropy sinks.

Using the first ten global modes reveals the main network of bidirectional causal interrelations among IP6 and PIP2 binding sites, envelope protein and myristoyl interaction sites, trimeric and further oligomerization sites of MA domains. The exclusion of the slowest mode discloses the subtler causal interactions. Please note that IP6 molecules were not considered explicitly in the calculations, which thus mostly reflect the intrinsic behavior of monomers and oligomers.

## Results

### Crystal structures of MA-IP6 complexes

**Figure 1:**
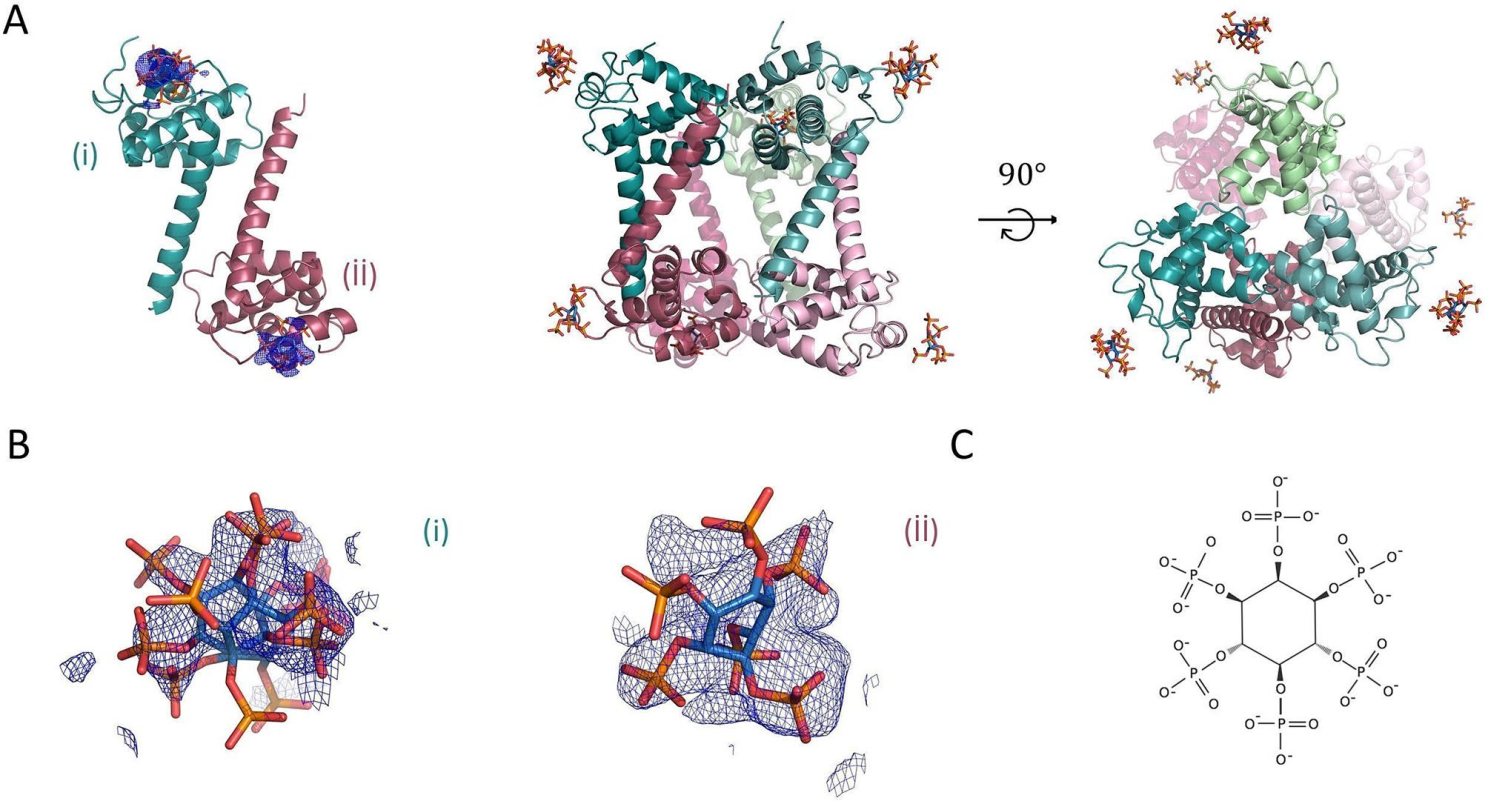
The overall structure of MA_IP6_R32 with symmetric units. (**a**) Chains A and B of MA_IP6_R32 structure in the asymmetric unit cell are colored in deep-teal and raspberry, respectively. Chain A and B symmetry mates are generated in *PyMOL* and are colored in pale green and light pink for the representation of symmetric units, respectively. Carbon, oxygen and phosphorus atoms of IP6 molecules are colored by sky blue, red and orange, respectively. (**b**) 2*F*o-*F*c simulated annealing-omit map is colored in blue mesh and shown at 2.5σ level within 5.0 Å from IP6. (**c**) Chemical structure representation of the *myo*-IP6 molecule.

**Figure 2:**
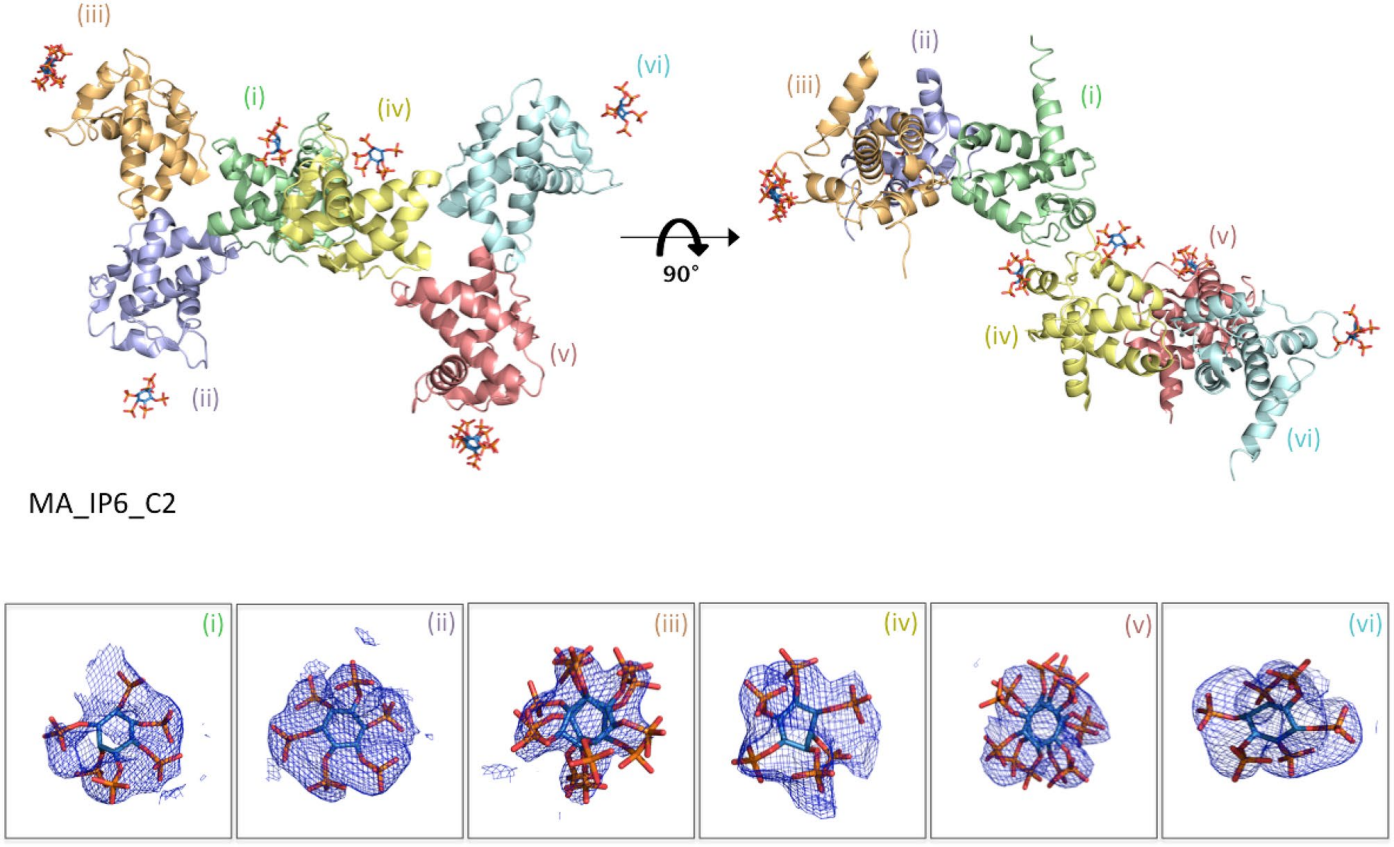
The overall structure of MA_IP6_C2 in an asymmetric unit. MA proteins in the asymmetric unit are shown by cartoon representation. Each chain is indicated by a different color and labeled with the respective color (pale green: chain (**i**), light blue: chain (**ii**), light orange: chain (**iii**), pale yellow: chain (**iv**), salmon: chain (**v**), pale-cyan: chain (**vi**). Carbon, oxygen and phosphorus atoms of IP6 are colored by sky-blue, red and orange, respectively. A 2*F*o-*F*c simulated annealing-omit map colored in blue mesh at 2.5σ level within 5.0 Å from IP6 is shown. For better view density maps derived from IP6 molecules are enlarged and shown in the insets.

**Figure 3:**
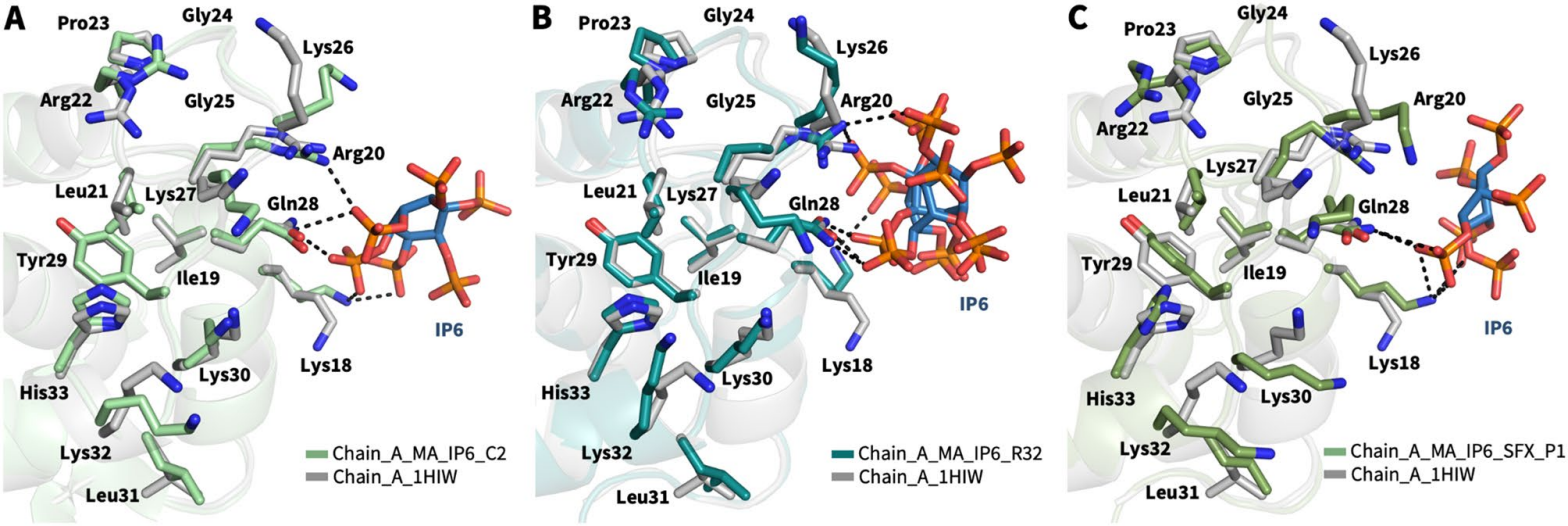
Superposition of MA domain structure (PDB ID: 1HIW) and our structures. (**a**) The chain A of our structure in green is superposed with chain A of MA domain (PDB ID: 1HIW) in gray, RMSD 0.279 Å. (**b**) The chain A of our MA_IP6_R32 structure in deep teal is superposed with chain A of MA domain (PDB ID: 1HIW) in gray, RMSD 0.273 Å. (**c**) The chain A of our MA_IP6_SFX_P1 structure in smudge is superposed with chain A of MA domain (PDB ID: 1HIW) in gray, RMSD 0.553 Å. The conformational changes on the residues in the HBR (18–33) are indicated and hydrogen bonds within the MA structures complex with IP6 are shown with dashed lines.

We employed X-ray crystallography to reveal the molecular mechanism of interaction between the MA domain and IP6 molecules. MA protein and IP6, which is the most abundant inositol in vivo, were co-crystallized. From three crystal forms, different crystal structures of MA complexed with IP6 (MA_IP6) were determined. Cryogenic MA_IP6_R32 structure is at 2.40 Å, Cryogenic MA_IP6_C2 extends to 2.72 Å, and ambient-temperature MA_IP6_SFX structure is determined at 3.5 Å resolution, respectively (Figs. 1 and 2, Supplementary Figs. 3 and 4). Our overall MA_IP6 complex structures are consistent with the previous NMR and crystal structures (PDB IDs: 2HMX and 1HIW, respectively) with the MA domain composed of 5 alpha-helices (Supplementary Table 2 and 3). Compared to the previous structures, a globular domain composed of the 1st to 4th helices did not show significant conformational change, whereas the C-terminal 5th helix did and will be further discussed later. Trimeric assembly of MA showed no significant overall structural differences between the MA_IP6 complexes and MA Apo form (PDB ID: 1HIW). However, a superposition of chains A of MA_IP6_C2, MA_IP6_R32 and MA_IP6_SFX_P1 with 1HIW form showed side-chain conformational changes within the HBR (18–33) of the N-terminus specifically residues Lys18, Arg20, Arg22, Lys26, Lys27, Gln28, Lys30, Leu31, Lys32, and His33 (Fig. 3). These conformational changes are involved in the interaction between MA and IP6 (Arg20, Gln28, Lys18) in our monomeric structure. Based on the density map of MA_IP6_C2, the *B*-factors of each IP6 molecule are notably high compared to MA (Fig. 3A and Supplementary Table 4). This suggests that the binding modes of IP6 molecules are not well-defined and that the affinities of MA with each IP6 varies. In our MA_IP6_R32 crystal form, the density map derived from IP6 was also observed at the same binding sites (Fig. 1). The observed differences of individual IP6 molecule interactions in each MA structure will be further explained below.

### IP6-binding sites of MA

**Figure 4:**
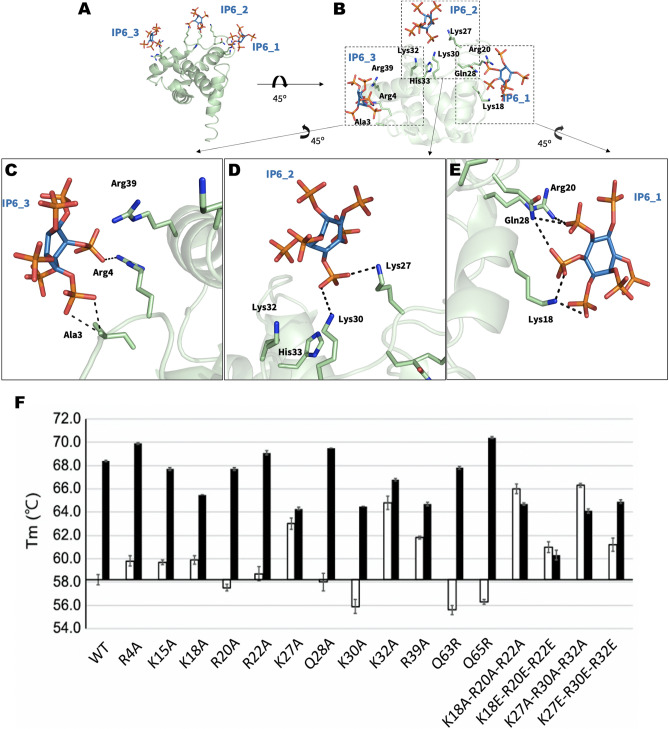
IP6-interacting amino acid residues of MA. (**a**) IP6 molecules near chain A and its symmetry mates of MA are shown in sky-blue and the side chain of amino acid residues in 5.0 Å is shown by sticks (pale green). (**b**) The three IP6 molecules are represented with their interacting residues on chain A. The interaction between (**c**) IP6_3 (**d**) IP6_2 (**e**) IP6_1 and chain A of the MA domain is shown in more detail. (**f**) DSF assay results of point mutations of MA in the presence (black bars) and in the absence of IP6 (white bars). Melting temperatures (Tm) of the wild-type and mutant proteins are indicated after normalization with wild-type protein Tm in the absence of IP6.

**Figure 5:**
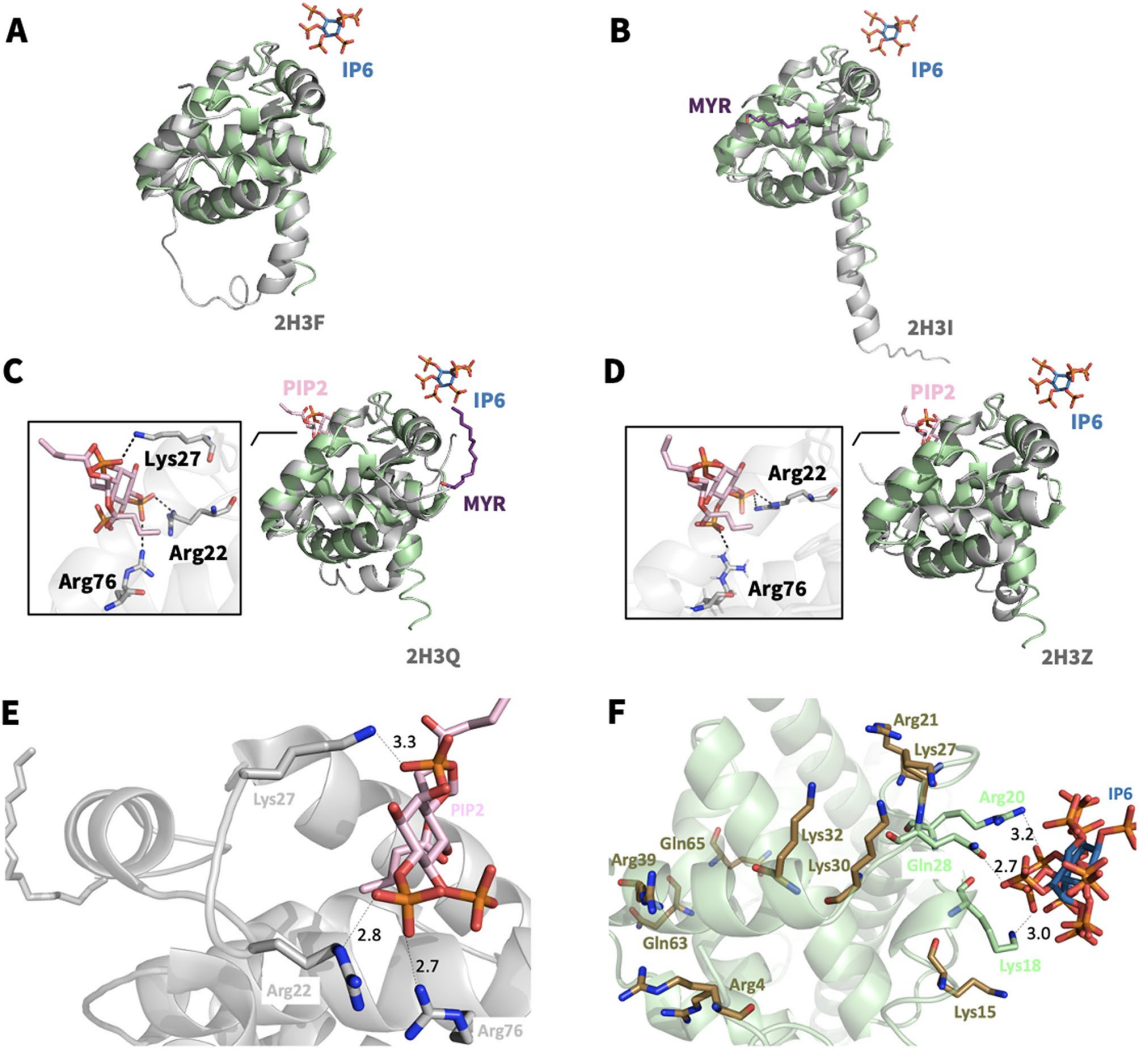
Structural comparison of MA_IP6_C2 with other MA complexes. Chain A of MA_IP6_C2 complex in green is superposed (**a**) with MA structure in gray (PDB ID: 2H3F), RMSD of 0.864 Å; (**b**) with MA structure that has myristoylation (purple) in gray (PDB ID: 2H3I), RMSD of 0.737 Å; (**c**) with MA structure that has myristoylation (purple) and PIP2 in gray (PDB ID: 2H3Q), RMSD of 0.742 Å; (**d**) with MA structure that has PIP2 in gray (PDB ID: 2H3Z), RMSD of 0.818 Å. The interaction between (**e**) IP6 and MA domain, (**f**) PIP2 and MA domain (PDB ID:2H3Q) is represented in more detail to equate the position of the corresponding molecules and angles more clearly. PIP2 is shown in color ‘light pink’, IP6 is shown in ‘sky-blue’, bound residues in ‘deep-olive’, rest of the highly basic region in ‘forest’, rest of the domain in ‘Gray’. Also, Lys27, Lys30, Lys32 and Arg39, Gln63 and Gln65 are shown to specify the regions mutated in DSF studies regarding the melting temperature. O is coded with red color, N with blue and P with orange. This figure shows that both PIP2 and IP6 bind to the MA domain within the highly basic region, however, the residues are different. Distances (within hydrogen bond boundaries) are also shown here in Angstrom. For IP6 binding, only the shortest distance between Gln28 and the IP6 is shown among 3 for clarity.

In the MA_IP6_C2 structure, a single chain of oligomeric MA interacts with three IP6 molecules, when considering symmetry mates of IP6. Three IP6 molecules were observed on chain A (Fig. 4A–E); one is on the asymmetric molecule of chain A (Fig. 5E, IP6_1), another is mainly in contact with chain D and visualized on chain A (Fig. 4D, IP6_2), and the last one is on the asymmetric molecule of chain A (Fig. 4C, IP6_3). Lys18, Arg20 and Glu28 formed hydrogen bonds with IP6_1. Lys27, Lys30, Lys32 and His33 are located close to IP6_2 and two hydrogen bonds were observed between Lys27, Lys30 and IP6_2. Ala3, Arg4 and Arg39 are near IP6_3 and Ala3, Arg4 interacts with this IP6 molecule. Additionally, when the MA_IP6_R32 structure was superposed with the MA_IP6_C2 structure, the binding position of IP6 molecules in each chain was detected in the same binding surface for both crystal forms (Supplementary Fig. 5). The interactions between IP6_1 and MA in the two crystal forms were analyzed to investigate differences of non-covalent interactions, but the same interacted residues were observed for both structures (Supplementary Fig. 6). To evaluate and dissect the contribution of these interactions, we employed Differential Scanning Fluorimetry (DSF).

### IP6 has an alternative binding site for MA non-overlapping with a PIP2 binding pocket

IP6 and PIP2 are both involved in the viral life-cycle as cofactors. Previous work suggests that IP6 has a similar function with PIP2 in the viral life-cycle (Supplementary Fig. 1)^25^. To test this, we have compared the MA binding sites for both IP6 and PIP2 molecules (Fig. 5 and Supplementary Table 4). We superposed our high-resolution X-ray structure of the MA_IP6_C2 complex with an existing PIP2 bound model (PDB ID: 2H3Q) (Fig. 5C, E and F). As a result, a comparison of MA_IP6_C2 structure with MA_PIP2 complexes indicated the different binding positions for PIP2 and IP6 within the HBR. For instance, PIP2 interacts with residues Arg22, Lys27, Arg76 (PDB ID: 2H3Q) (Fig. 5C); and Arg22, Arg76 (PDB ID: 2H3Z) (Fig. 5D) respectively. Whereas IP6_1 interacts with Lys18, Arg20 and Gln28 through the hydrogen bonds in our MA_IP2_C2 structure. Furthermore, symmetry mates of chain A of MA showed that IP6 (IP6_2 and IP6_3) can bind to MA with different residues within the HBR, leading to a potential competition as a cofactor between IP6 and PIP2 (Fig. 4). One of the residues Lys27 which interacts with both IP6 and PIP2 with Myr presence, this competition may play an important role during the envelope incorporation process for making equilibrium between MA assembly on the membrane by IP6 and anchoring by PIP2-Myr interactions. In addition, the different conformation states on the C-terminus were observed between MA_IP6_C2 and MA_IP6_R32 (Supplementary Figs. 5 and 6). Our structures demonstrate that IP6 binds to MA from the nearby region but not the same amino acid residues involved in PIP2 binding (Fig. 5). These observations collectively suggest that both IP6 and PIP2 can simultaneously bind the MA. This intricate interplay of these binding events of these two host molecules may orchestrate the membrane binding and assembly either simultaneously or sequentially.

### DSF assay

For understanding the underpinnings of possible binding sites of IP6 on MA domain several single and multiple mutants were performed and then melting temperatures (Tm) of the wild-type (WT) MA domain and its mutants were determined by DSF (Fig. 4F). In addition to that, the effect of IP6 presence and absence on the WT and its mutants were observed. In the absence of IP6, R4A, K15A, R20A, R22A, Q28A and Q65R single mutations affected the Tm less than 2 °C while K18A, K27A, K30A, K32A, R39A and Q63R single mutations caused the notable change. Among them, K18A, K27A, K32A and R39A mutations increased the Tm according to WT-MA whereas, K30A and Q63R mutations decreased the Tm. We preferred to use ΔTm (ΔTm: Tm difference between the presence and absence of IP6) in comparisons because there is more than one factor affecting Tm in mutations in the presence of IP6 (Supplementary Fig. 7). K18A, K27A, K30A and R39A mutations decreased the Tm of MA protein in the presence of IP6, while Q65R mutation increased it. To test these residues’ combined effects, we combined these mutations and formed new triple mutants and determined their Tm. We generated two mutation sets based on these single mutation results: K18-R20-R22 and K27-K30-K32. These amino acid sets were mutated to alanine and aspartate for each mutant combination. K18A-R20A-R22A, K18E-R20E-R22E, K27A-K30A-K32A and K27E-K30E-K32E triple mutants have higher Tm than WT-MA domain in the absence of IP6. In the presence of IP6 within the DSF reaction medium, K18A-R20A-R22A, K18E-R20E-R22E and K27A-K30A-K32A triple mutations were observed to have a decreased MA Tm than the wild type. Single-residue mutants K27A, K32A, R39A and all the multiple mutants show little to no change between the presence and absence of IP6. Based on these findings, we can deduce that the mutations on IP6 binding sites inhibit IP6 interaction with MA. Finally, we conclude that presence of IP6 enhances the thermal stability of MA protein.

### Dynamic causal interrelations in the assembly of MA

**Figure 6:**
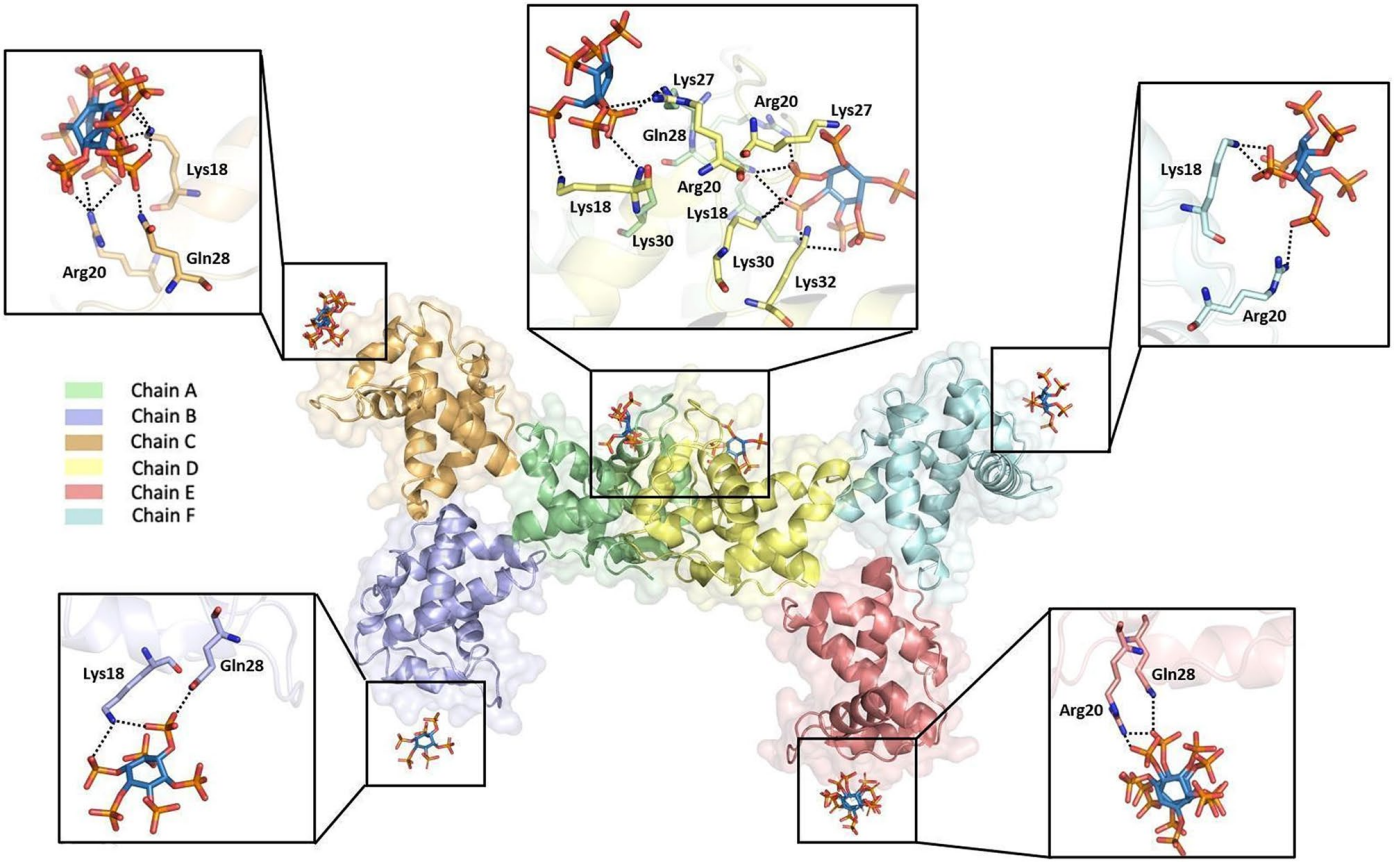
Representation of interactions on MA_IP6_C2 structure. The oligomeric structure of MA carries several IP6 molecules that interact with different residues between two trimeric proteins according to single trimer and IP6 interactions. Each chain is colored by individual colors as indicated previously. Carbon, oxygen and phosphorus atoms of IP6 are colored by sky-blue red and orange, respectively. The polar contacts between IP6 molecules and residues of MA oligomer are shown with dotted lines.

We explored time-dependent structural fluctuations and dynamic causal residue interrelations in the hierarchical assembly of HIV-1 MA domain through its monomeric, trimeric, hexameric and dodecameric structures by utilizing their global dynamic modes. GNM based transfer entropy TE calculations disclosed an interplay of dynamic allosteric interactions among IP6, PIP2, membrane (envelope protein and myristoyl) and trimeric and higher oligomeric interaction sites, presented in Fig. 6 & Supplementary Figs. 8–12 and detailed in Supplementary Results, suggesting a dynamic order in the oligomerization of the MA domain.

### Order of events predicted by GNM-based transfer entropy calculations

**Figure 7:**
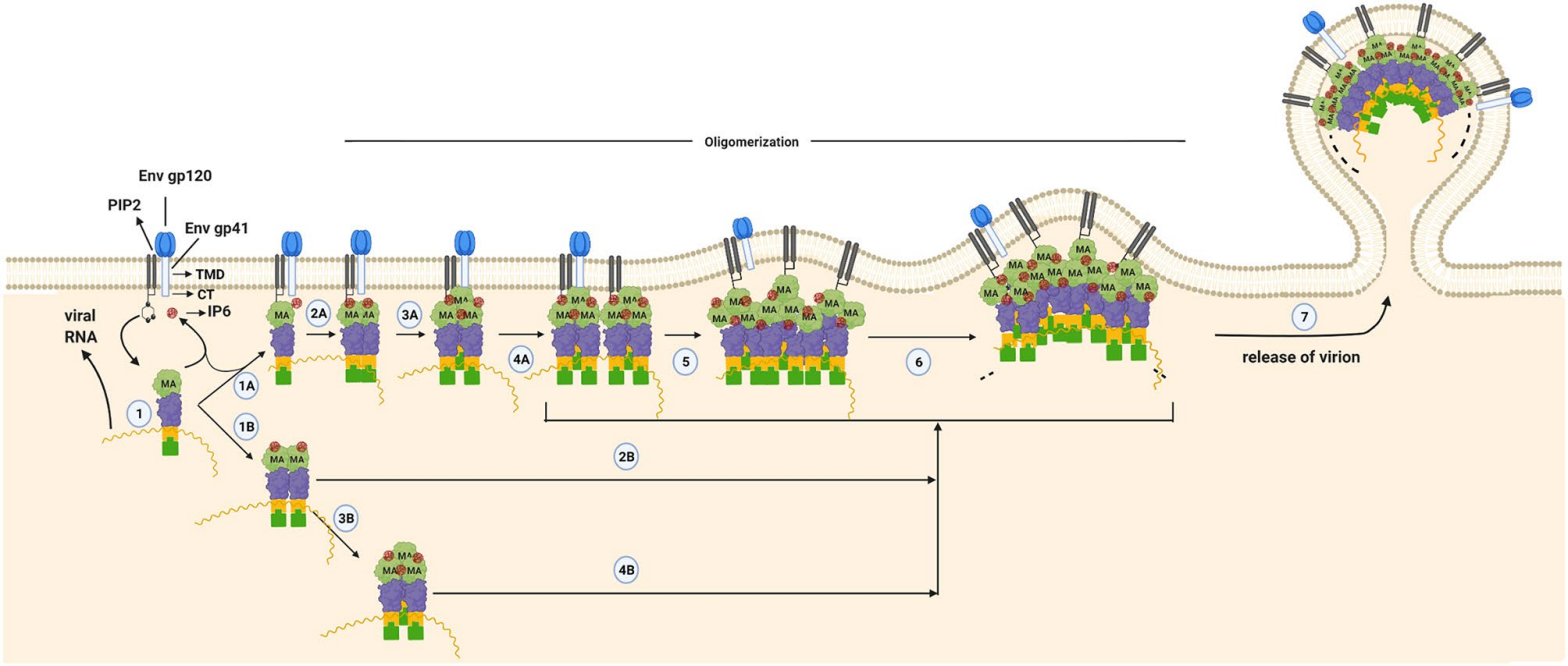
A plausible order of events for Pr55^Gag^ oligomerization. MA domain of Pr55^Gag^ interacts with PIP2 and IP6 to orchestrate Pr55^Gag^ integration into the host plasma membrane (1) The interaction process is shown in the figure by labeling as A and B to represent plausible alternate kinetic pathways. According to this model, the process can be proceeded by dimerization or trimerization of the monomer form of Pr55^Gag^ (1B & 3B, respectively). The formed dimer or trimer structure in the cytosol can be involved in the oligomerization process by the contribution of PIP2 and IP6 (2B&4B) and the progression of the higher-order oligomerization can continue in the membrane (5, 6 & 7). Besides, direct binding of the monomer form of Pr55^Gag^ to the membrane (1A) may lead to further oligomerization (2A- 4A) for the integration of Pr55^Gag^. IP6 binding (one-per-monomer) facilitates trimerization, possibly via trimerization sites, affecting further oligomerization and envelope/membrane binding through Env gp41 penetration to the cellular membrane. Together with this, PIP2 binding might trigger interactions with the envelope protein, while IP6 binding and the status of trimerization may affect membrane interactions. Oligomerization sites with IP6 and PIP2 binding might also affect trimer stability; this distinct bidirectional causality along with membrane and myristoyl interactions likely ensures the high order assemblies with optimum parameters. **TMD**: Transmembrane domain. **CT**: Cytoplasmic tail.

To identify predominant directional communication paths, an average of the ten slowest GNM modes of motion were used while an average of two to ten slowest dynamic modes were used to disclose subtler interaction paths. The presence of the slowest mode suggests a more collective and dominant dynamic behavior. Figure 7 features a plausible order of events as follows. **﻿1–3.** IP6 binding on sites Lys18, Arg20 and Gln28 of the monomer facilitates trimerization, possibly via trimerization site I (42–48) first, since its entropy receiving role from the IP6 binding sites is embedded in the slowest mode. For trimerization site II (64–72), similar facilitation by IP6 (Arg20) appears when we remove the slowest mode. The occupation of either trimerization sites might dynamically affect each other since there is evident information entropy transfer between these two sites of the monomer. On the other hand, PIP2 binding might trigger interactions with the envelope protein initially via Arg76, which is a prominent entropy source to the envelope interaction sites (Leu12, Glu16 and Leu30) in the presence of the slowest mode. As PIP2 and MA’s Arg76 is farther from the IP6 site, it could potentially generate a unique PIP2-specific response in the presence of PIP2. IP6 binding and trimerization state of the monomer at trimerization site II (bound to another monomer or not) might also affect envelope protein interactions with the entropy transfer from the first two sites to the latter in the presence of the slowest mode. **4.** The interaction status with the envelope protein, hence membrane, affects further oligomerization. Envelope interaction sites drive the movement of the oligomerization region residues Arg20, Lys27, Gln28 and Lys30 overlapping with IP6 sites in the slowest mode of the trimer. This might indicate that the presence of a membrane affects oligomerization in higher order (as hexamer or higher forms). **5 & 6.** PIP2 via Arg76 and trimerization sites might have a role in higher state oligomerization since these sites drive the movement of oligomerization sites in the slowest mode of the hexamer. On the other hand, oligomerization state (bound to another trimer or not) and IP6 and PIP2 binding might also affect trimer stability. The higher oligomerization site residues (Arg20, Lys27, Gln28 and Lys30) also transfer entropy to trimerization sites without the slowest mode, i.e. in average two to ten slow modes, of the hexamer. This bidirectional communication might ensure the organization of higher-order assemblies with optimum parameters, such as curvature radius, to enable the eventual formation of proper capsids. **After 6.** IP6-monomer binding that occurs at a greater than one-to-one ratio might affect further oligomerization and envelope/membrane binding. Extra IP6 binding sites (for IP6_2 and IP6_3) were seen in higher oligomerization states affecting oligomerization sites and envelope protein binding sites. Additionally, interactions among the trimers via trimerization sites affect the further oligomerization possibly via adjusting the trimer components (assuming flexible interactions instead of a rigid behavior for trimers) in response to the need of relevant higher-order oligomerization state.

## Discussion

Different HIV-1 proteins, enzymes and metabolic steps from the first contact with the host to the production of new viral particles have crucial roles in the HIV-1 life cycle and have been potential drug targets. Pr55^Gag^ protein regulates HIV-1 assembly, budding and maturation of virions. Working with this essential protein and its complexes may provide a further understanding of the formation of virions^4^. Pr55^Gag^ contains MA, CA, NC, and p6 domains, where the MA domain regulates Pr55^Gag^ membrane binding and assembly^49,50^. Given the key importance of the MA domain in the virion cycle, inhibition of this protein may provide an effective treatment against HIV and makes it an important drug target^18,51^.

Previous studies revealed the physical interaction between both PIP2-MA and IP6-MA *in vitro*^10,25,52–55^. It is shown that inositol phosphates are crucial cofactors involved in the complex HIV-1 assembly process^7,25^. The need for the presence of IP6 in the viruses, even in "encapsidation" alone, is a milestone for HIV virion formation. On the other hand, IP6 has a strong influence on not only CA but also MA trimerization and has a key role in assembly and encapsidation^25^. Additionally, IP6 has been shown to interact with MA at the highest level among inositol phosphates species^51^. The requirement of IP6 in the different steps of the viral life-cycle makes IP6 the target of the study. Therefore, any high-resolution structural information about the IP6-MA complex will provide invaluable details of the assembly process. Our study demonstrates that IP6 and PIP2 binding is not mutually exclusive, but rather they co-localize on non-overlapping binding site residues and alter the conformation of MA domain on the plasma membrane. The C-terminus of the MA domain is crucial for CA domain binding. Additionally, the conformation of Gag was determined with various of conformational states by different oligomeric structures including trimers and hexamers (Supplementary Fig. 5). The different conformations of C-terminal by trimer or hexamer MA with IP6 interaction can induce an overall conformational change of Gag protein assembly by squeezing and bending the proteins’ opposite terminals. Our hexamer structure with IP6 binding displays these possible conformations (Supplementary Fig. 13).

Our high-resolution MA_IP6_C2 structure demonstrates critical interactions of IP6 with MA explains how IP6 may favor trimeric state (Fig. 6). It may also provide an explanation for the previous observation that the presence of IP6 favors the higher-order oligomeric state of MA trimers^25^. These interactions are largely mediated through H-bonds by the basic side chain residues Lys and Arg together with polar uncharged Gln residues. Importantly, two nearby IP6 molecules are present at the site where the two MA trimers forming the hexamer are involved in pivotal intermolecular interactions. Based on crystal packing and contact patterns, the interaction between IP6 and MA at the selected trimer-trimer interaction site is mediated by the chain A residues Arg20, Lys27, Gln28, Lys30 and the chain D residues Lys18, Arg20, Lys30, Lys32 (Fig. 6). Lys27 is important for the comparison of IP6 and PIP2 binding because this residue interacts with both of these molecules, revealing a plausible mechanism for the connectivity between PIP2 and IP6 (Figs. 5 and 6). This result suggests that after the induction of Gag oligomerization, IP6 attached to this region may be replaced with PIP2 when Gag reaches the membrane; and IP6 binding helps the MA domain for higher-order oligomerization, showing that IP6 and PIP2 binding may be compatible. Besides, chain B, C, E and F form interactions with IP6 molecules by Lys18, Arg20 and Gln28 residues in varying binding angles, distances and conformations (Fig. 6). This result may show that H-bond interactions gave specificity but electrostatic interaction of side chains resulted in different angles, distances, and conformations for IP6 and MA binding in each of the trimers. The main distinction of the MA trimer dimerization interface is the involvement of three different amino acid interactions at Lys27, Lys30 and Lys32 besides monomer level MA and IP6 interactions. We have noticed that the residues mutated in DSF analysis (Fig. 4F) are not directly related to the monomeric level binding of MA to IP6; therefore, they either have a role in oligomerization (likely Lys27, Lys30 and Lys32) or structural stability of MA domain (Gln63 and Gln65). Another possible explanation for this situation is that there is more than one location where IP6 can bind and interact with the MA domain, each site responsible for different kinetic pathways in the assembly process (Figs. 4 and 6).

Our data explain the previous in vitro results in which they showed that in the presence of IP6, MA enhances the formation of trimers^25^. Acidic IP6 molecules occupy corners of the MA protein promoting the protein trimerization from the sides by attracting their basic charges (Supplementary Figs. 14 and 15). Trimerization of the MA may provide more stability and binding of IP6, thereby increasing the Tm of the MA protein. The IP6 molecules present in our structures allowed us to provide a model for high-order oligomerization of MA and suggest a role for IP6 in these multistep structural rearrangement events (Supplementary Fig. 13). It was shown that not delivery of Gag to the membrane but virion assembly during budding is perturbed by reduced cellular IP6 levels^22^. This may suggest that MA-IP6 complex occurs during virion assembly and budding. Here, we suggest a new model for the formation of higher-degree MA oligomers, induced and stabilized by IP6 binding. IP6 binding stabilizes the expanded conformations of an oligomeric and more organized state on the plasma membrane (Supplementary Fig. 13B–D). Although the role of IP6 on budding mechanisms is still unclear, it is possible that the interactions of PIP2 and IP6 binding can occur simultaneously in this mechanism, since they have non-overlapping binding sites on MA.

To explain the effect of IP6 on the MA protein, its membrane interaction and higher-order oligomerization, causal dynamic interactions in different oligomerization states of MA were explored by using transfer entropy TE calculations for directional allosteric interactions (Supplementary Fig. 8–12). While some residues convey information as entropic sources, some receive it as entropic sinks. With their interchangeable roles as entropic sources and sinks, IP6/PIP2 binding sites and trimeric interfaces of MA display a dynamic interplay among each other, resulting in a bidirectional information flow manifested with the dissection of slow modes of motion/informational entropy. In the trimer, the interactions between the envelope protein and myristoyl molecules are the major entropic sources. The dynamic fluctuations of these sites will possibly lead to the conformational and/or dynamic changes in the various regions of the trimer including trimerization sites along with IP6 and PIP2 binding sites. At this early oligomerization step as a trimer, envelope protein and myristoyl interaction sites might be more important due to the membrane attachment, relaying information regarding the membrane presence toward the trimerization regions along with IP6 and PIP2 binding sites. On the other hand, a similar bidirectional causality among IP6 and PIP2 sites and trimeric interfaces (interfaces of the MA domains) are observed in both structures of the hexamers despite their distinct structural organizations. Here, PIP2 and trimeric interfaces appear as the main drivers (i.e., entropic sources) with IP6 causing subtler allosteric interactions. In conclusion, allosteric signals are bidirectional, and the dynamic information is conveyed in opposite directions by recruiting different modes of motion. These movements form a dynamic repertoire for the protein. It provides functional conformation and dynamical changes in response to the perturbations and environment by exerting certain relevant modes of motion. Those results verified the previous findings of IP6 effect on MA trimerization; PIP2 and myristoyl interactions on membrane attachment supported the concepts of IP6 on higher-order oligomerization; and alteration of membrane localization and binding with PIP2. Still, further molecular dynamics studies must be carried out for higher degree oligomerization of MA in the presence of IP6. These studies can be divided into two subtopics: (1) trimer dimerization; (2) simulation of dissecting different binding modes of IP6 on MA and its effect on the stability of the larger oligomeric state.

Dynamic interaction of PIP2 and IP6 on MA is crucial for promoting Gag localization to the plasma membrane during the viral maturation. That is why, targeting MA through these two small molecules carries significant importance for molecular design of next generation anti-HIV-1 agents to block membrane localization of Pr55^Gag^ and virion formation. The data presented here adds a new structural perspective to the predicted interaction between MA-IP6 for further studies. Our suggested model reveals potential new target sites to develop novel therapies for the treatment of HIV-AIDS.

## Supplementary Information

Supplementary Information.

## References

[1] Chinchar, V. G. Replication of Viruses. *Encycl. Virol.* 1471–1478 (1999).

[2] Walker, B. & Mcmichael, A. The T-Cell Response to HIV. 1–19 (2012). 10.1101/cshperspect.a007054 PMC3543107 23002014

[3] Freed EO (2015). HIV-1 assembly, release and maturation. Nat. Rev. Microbiol..

[4] Sundquist, W. I. & Kra, H. HIV-1 Assembly, Budding, and Maturation. 1–24 (2012). 10.1101/cshperspect.a006924 PMC3385941 22762019

[5] Ghanam RH, Samal AB, Fernandez TF, Saad JS (2012). Role of the HIV-1 matrix protein in Gag intracellular trafficking and targeting to the plasma membrane for virus assembly. Front. Microbiol..

[6] Gaines CR (2018). HIV-1 matrix protein interactions with tRNA: implications for membrane targeting. J. Mol. Biol..

[7] Dick RA (2018). Inositol phosphates are assembly co-factors for HIV-1. Nature.

[8] Massiah MA (1994). Three-dimensional structure of the human immunodeficiency virus type 1 matrix protein. J. Mol. Biol..

[9] Tang C (2004). Entropic switch regulates myristate exposure in the HIV-1 matrix protein. Proc. Natl. Acad. Sci. U. S. A..

[10] Ono A, Ablan SD, Lockett SJ, Nagashima K, Freed EO (2004). Phosphatidylinositol (4,5) bisphosphate regulates HIV-1 Gag targeting to the plasma membrane. Proc. Natl. Acad. Sci. U. S. A.

[11] Freed EO, Englund G, Martin MA (1995). Role of the basic domain of human immunodeficiency virus type 1 matrix in macrophage infection. J. Virol..

[12] Murray PS (2005). Retroviral matrix domains share electrostatic homology: Models for membrane binding function throughout the viral life cycle. Structure.

[13] Alfadhli A (2016). Trimer enhancement mutation effects on HIV-1 matrix protein binding activities. J. Virol..

[14] Sanford B (2014). Deletions in the fifth alpha helix of HIV-1 matrix block virus release. Virology.

[15] Freed EO, Orenstein JM, Buckler-White AJ, Martin MA (1994). Single amino acid changes in the human immunodeficiency virus type 1 matrix protein block virus particle production. J. Virol..

[16] Freed EO (1998). HIV-1 gag proteins: diverse functions in the virus life cycle. Virology.

[17] Zhou W, Parent LJ, Wills JW, Resh MD (1994). Identification of a membrane-binding domain within the amino-terminal region of human immunodeficiency virus type 1 Gag protein which interacts with acidic phospholipids. J. Virol..

[18] Tateishi H (2017). A clue to unprecedented strategy to HIV eradication: ‘Lock-in and apoptosis’. Sci. Rep..

[19] Saad JS (2006). Structural basis for targeting HIV-1 Gag proteins to the plasma membrane for virus assembly. Proc. Natl. Acad. Sci. U. S. A..

[20] Campbell S (2001). Modulation of HIV-like particle assembly in vitro by inositol phosphates. Proc. Natl. Acad. Sci. U. S. A..

[21] Letcher AJ, Schell MJ, Irvine RF (2008). Do mammals make all their own inositol hexakisphosphate?. Biochem. J..

[22] Mallery DL (2019). Cellular IP6 levels limit HIV production while viruses that cannot efficiently package IP6 are attenuated for infection and replication. Cell Rep..

[23] Siddhartha AKD (2007). Interactions between HIV-1 gag molecules in solution: an inositol phosphate-mediated switch. Nat. Institutes Heal..

[24] Sierra-Aragón S, Walter H (2012). Targets for inhibition of HIV replication: entry, enzyme action, release and maturation. Intervirology.

[25] Alfadhli A (2019). Analysis of HIV-1. J. Virol..

[26] Hiraki M (2006). Development of an automated large-scale protein-crystallization and monitoring system for high-throughput protein-structure analyses. Acta Crystallogr. Sect. D Biol. Crystallogr..

[27] Kabsch W (2010). XDS. Acta Crystallogr. Sect. D Biol. Crystallogr..

[28] Evans P (2006). Scaling and assessment of data quality. Acta Crystallogr. Sect. D Biol. Crystallogr..

[29] Evans PR, Murshudov GN (2013). How good are my data and what is the resolution?. Acta Crystallogr. Sect. D Biol. Crystallogr..

[30] Vagin A, Teplyakov A (1997). MOLREP: an automated program for molecular replacement. J. Appl. Crystallogr..

[31] Murshudov GN, Vagin AA, Dodson EJ (1997). Refinement of macromolecular structures by the maximum-likelihood method. Acta Crystallogr. Sect. D Biol. Crystallogr..

[32] Afonine PV (2012). Towards automated crystallographic structure refinement with phenix.refine. Acta Crystallogr. Sect. D Biol. Crystallogr..

[33] Emsley P, Lohkamp B, Scott WG, Cowtan K (2010). Features and development of Coot. Acta Crystallogr. Sect. D Biol. Crystallogr..

[34] Krissinel E, Henrick K (2007). Inference of macromolecular assemblies from crystalline state. J. Mol. Biol..

[35] Ciftci HI (2019). Serial femtosecond X-ray diffraction of HIV-1 gag MA-IP6 microcrystals at ambient temperature. Int. J. Mol. Sci..

[36] Herrmann S (2013). CSPAD-140k: A versatile detector for LCLS experiments. Nucl. Instruments Methods Phys. Res. Sect. A Accel. Spectrometers, Detect. Assoc. Equip..

[37] Sierra RG (2016). Concentric-flow electrokinetic injector enables serial crystallography of ribosome and photosystem-II. Nat. Methods.

[38] Damiani D (2016). Linac coherent light source data analysis using psana. J. Appl. Crystallogr..

[39] Thayer J (2017). Data systems for the Linac coherent light source. Adv. Struct. Chem. Imaging.

[40] White TA (2012). CrystFEL: A software suite for snapshot serial crystallography. J. Appl. Crystallogr..

[41] White TA (2019). Processing serial crystallography data with crystFEL: a step-by-step guide. Acta Crystallogr. Sect. D Struct. Biol..

[42] Mallery DL (2018). IP6 is an HIV pocket factor that prevents capsid collapse and promotes DNA synthesis. Elife.

[43] Huynh K, Partch CL (2015). Analysis of protein ligand-receptor binding by photoaffinity cross-linking. Curr. Protoc. Protein Sci..

[44] Bahar I, Atilgan AR, Erman B (1997). Direct evaluation of thermal fluctuations in proteins using a single-parameter harmonic potential. Fold. Des..

[45] Haliloglu T, Bahar I, Erman B (1997). Gaussian dynamics of folded proteins. Phys. Rev. Lett..

[46] Hacisuleyman A, Erman B (2017). Causality, transfer entropy, and allosteric communication landscapes in proteins with harmonic interactions. Proteins Struct. Funct. Bioinforma..

[47] Acar B (2020). Distinct allosteric networks underlie mechanistic speciation of ABC transporters. Structure.

[48] Bahar I, Cheng MH, Lee JY, Kaya C, Zhang S (2015). Structure-encoded global motions and their role in mediating protein-substrate interactions. Biophys. J..

[49] Murakami T (2008). Roles of the interactions between Env and Gag proteins in the HIV-1 replication cycle. Microbiol. Immunol..

[50] Dubois N (2018). The C-terminal p6 domain of the HIV-1 Pr55Gag precursor is required for specific binding to the genomic RNA. RNA Biol..

[51] Tateishi H (2014). Design and synthesis of lipid-coupled inositol 1,2,3,4,5,6-hexakisphosphate derivatives exhibiting high-affinity binding for the HIV-1 MA domain. Org. Biomol. Chem..

[52] Mercredi PY (2016). Structural and molecular determinants of membrane binding by the HIV-1 matrix protein. J. Mol. Biol..

[53] Saad JS (2008). Structure of the myristylated human immunodeficiency virus type 2 Matrix protein and the role of phosphatidylinositol-(4,5)- bisphosphate in membrane targeting. J. Mol. Biol..

[54] Ehrlich LS, Medina GN, Carter CA (2011). ESCRT machinery potentiates HIV-1 utilization of the PI(4,5)P 2-PLC-IP3R-Ca2+ signaling cascade. J. Mol. Biol..

[55] Ganser-Pornillos BK, Yeager M, Sundquist WI (2008). The structural biology of HIV assembly. Curr. Opin. Cell Biol..

