# Supplementary material for: Structural insight into host plasma membrane association and assembly of HIV-1 matrix protein
Source: Sci Rep. 2021 Aug 4;11:15819. doi: 10.1038/s41598-021-95236-8 (PMC8339130; doi:10.1038/s41598-021-95236-8)
Supplement: Supplementary file 1 — Supplementary Information. [file 41598_2021_95236_MOESM1_ESM.pdf]

Supplementary Results:

Dynamic causal interrelations in monomeric, trimeric, hexameric and dodecameric structures of MA domain

Monomer (MA_IP6_C2)

Supplementary Figure S6 displays the causal interrelation of residue pairs in average ten (Figure S6A) and average two to ten (Figure S6B) slowest dynamic modes of the monomer, pointing to interactions among IP6 and PIP2 binding sites, trimerization sites I & II and envelope and myristyl interaction sites. The casual interrelations are rather promiscuous, yet some distinct causal interrelations are still observed as follows.

IP6 might be the major player in the monomer state of MA as the topology imposes as entropy sources the recruit of all three IP6 binding sites Lys18, Arg20 and Gln28 in the most global mode as well as Arg20 in average two to ten slowest dynamic modes. IP6 binding possibly facilitates trimerization via trimerization sites I (42-48) and II (64-72) with and without the slowest dynamic mode, respectively. The occupation of either trimerization site might also dynamically affect each other as shown by the information transfer TE between the two. IP6 binding and trimerization status (bound to another monomer or not) at trimerization site II might also affect envelope protein (Alfadhli, 2016) and myristoyl (Saad, 2007) interactions. For illustration, the causal interrelations of Lys18 and Arg20 (**Supplementary Figures S6A and S6B**, respectively) of IP6 binding sites were shown on the monomer (on the right).

On the other hand, different PIP2 sites transfer information via using distinct dynamic modes (Arg76 in average ten and Arg22 in average two to ten slowest dynamic modes; **Supplementary Figure S6)** to entropy receivers/sinks indicating a more delicate communication for PIP2. PIP2 binding via Arg76 might trigger interactions predominantly with the envelope protein while also affecting myristoyl interaction sites. This likely generates a unique PIP2 specific response by recruiting a residue farther than the IP6 site.

Trimer (MA_IP6_C2)

Supplementary Figure S7 displays the causal interrelation of residue pairs in average ten slowest

dynamic modes of the trimer. While all constituent chains behave similar to each other, entropy sources cluster in the vicinity of IP6 binding sites on these chains (yet shown only on chain A for simplicity). Among the entropy sources, envelope protein (Leu13, Glu17, Leu31, Val35 and Glu99) and myristoyl (Val7, Ile34, Val35, Leu51 and Glu52) interaction sites were both seen to drive the movement of trimerization sites I (Glu42-Pro48) and II (Leu64-Ser72) along with IP6 (Arg20, Gln28) and PIP2 (Arg22, Lys27 and Arg76) binding sites (also overlapping with higher oligomerization sites Arg20, Lys27, Gln28 and Lys30 than trimeric form). As exemplified on the right of Figure S7, the causal effects of the envelope-protein-interacting residue Leu13 (chain A) are distinctive as shown particularly at trimerization sites I and II. This implies that the interaction status with the envelope protein in collaboration with IP6 binding might trigger the oligomerization in high complexity. When the slowest dynamic mode is removed, a rather promiscuous causal interrelation appears (not shown). This might emphasize the role of the slowest dynamic mode in directing distinct causal signals from envelope and myristoyl interaction sites toward the IP6 and PIP2 binding sites along with both trimerization sites.

Additionally, if only five slowest instead of ten slowest dynamic modes are considered, the entropy sources would include also IP6 binding sites (Lys18 and Arg20) in addition to nearby sites (data not shown); overall indicating an important dynamic role for IP6 binding or nearby residues in initiating and facilitating the trimeric and higher oligomeric assembly process.

Hexamer (MA_IP6_C2)

Supplementary Figure S8A displays the causal interrelation for each residue pair in average ten slowest dynamic modes of the hexamer. The outmost chains C and F work as strong entropy sources in general, followed by chains B and E. On the other hand, IP6 binding sites of chains A and D overlapping with the higher oligomerization sites are responsible for trimer dimerization, mostly acting as entropy sinks and receiving the dynamic signal. Specifically, PIP2 (Arg76) binding site and trimerization sites I (Glu42-Pro48) and II (Leu64-Ser72) are the main entropic sources on the hexamer, which drive the movement of IP6 binding sites and their C-terminal neighbors (Lys18, Arg20, Gln28 and Asp93-Asp96 in black ellipses). As exemplary cases, the causal effects of Ala45 and Arg76 (chain B) clearly demonstrate the dynamic driving capacity of these residues on the movements of IP6 binding and nearby residues in the hexamer.

Supplementary Figure S8B, on the other hand, shows the causal interrelation for each residue pair in average two to ten slowest dynamic modes of the hexamer. IP6 and PIP2 binding sites along with myristoyl interaction sites appear as main entropy sources that drive the movement of trimerization sites I (Glu42-Pro48) and II (Leu64-Ser72). The entropy source residues Lys18 and Arg22 (chain A) exemplify IP6 and PIP2 binding sites driving the movement of both trimerization sites. Interesting to note here is that the distinct bidirectional causality could be observed with the dissection of information entropy TE into different dynamic mode sets, enabling to disclose both directions of allosteric signaling between IP6 binding and trimerization sites. On the other hand, PIP2 binding sites appear as a common player in both directions of the allosteric signaling between IP6 binding, trimerization and oligomerization sites. Conclusively, the emerging interplay between distinct interface residues (of monomers and trimers) might regulate the higher-order assembly of monomers.

Hexamer (MA_IP6_R32)

Supplementary Figure S9A displays the causal interrelation for each residue pair in an average ten slowest dynamic modes of the hexamer. While all constituent chains behave similarly to each other, PIP2 binding site Arg76 and trimerization sites I (Glu42-Pro48) and II (Leu64-Ser72) are the main entropic sources on each chain. These drive the movement of IP6 binding regions and their C-terminal neighbors (Lys18, Arg20, Gln28 and Asp93-Asp96) along with myristoyl interaction sites (Leu51, Glu52). As an example, the causal effects of Ala45 and Arg76 (chain A) indicate the driving role of these residues on IP6 and nearby residues. The second figure on **Supplementary Figure S9B,** on the other hand, shows the causal interrelation for each residue pair in average two to ten slowest dynamic modes of the hexamer. When the slowest dynamic mode is discarded, IP6 and PIP2 (1,3) binding and myristoyl (2) interaction sites on each chain (shown on chain B only for simplicity) are seen to drive the movement of trimerization sites I (Glu42-Pro48) and II (Leu64-Ser72). The affected residues of Lys18 and Arg22 (chain B) exemplifies IP6 and PIP2 binding sites as driving the movement of both trimerization sites. PIP2 binding sites appear as common players in both directions of the allosteric signaling between IP6 binding and trimerization sites. These observations suggest that IP6 and PIP2 can affect each other and the protein differently. The intricate interplay of these two ligand binding events may

orchestrate the membrane interactions and assembly.

The role of trimerization site dynamics is so robust in causal interactions of the hexamer that even different configurations of the hexamer result in a similar pattern of directional information flow orchestrated among the same regions.

Dodecamer (MA_IP6_P1).

Supplementary Figure S10 shows the causal interrelation for each residue pair in average ten slowest dynamic modes of the dodecamer (MA_IP6_P1). As being the utmost chains, G, H and I are the main entropic sources of the dodecamer. Trimerization sites I and II along with myristoyl interaction and higher state IP6 binding sites behave as driver residues on multiple chains. These sites mainly affect IP6 and PIP2 binding sites along with envelope protein interaction sites. Only on chain K (larger cumulative net transfer entropy than zero), IP6 and PIP2 binding sites act as entropy sources for the other IP6 and PIP2 binding sites on chains A, B, C. As exemplary cases, the causal interrelations of Arg4 (a higher oligomer IP6 binding residue on chain K) and Leu68 (located at trimerization site II on chain J) were shown. With the exclusion of the slowest dynamic mode, chains D, E and F are the main entropic sources, followed by chains J, K, L (data not shown). With or without the slowest mode, trimerization sites I and II along with myristoyl interaction and higher state IP6 binding sites behave as driver residues on multiple chains. These sites mainly affect IP6 and PIP2 binding sites along with envelope protein interaction and trimerization sites of the other trimers.

In summary, more than one IP6 binding per-monomer might affect further oligomerization and envelope/membrane binding. Trimerization sites affect further oligomerization possibly via adjusting the trimer components (assuming flexible interactions among constituent monomers instead of a rigid behavior for the trimers) in response to the need for relevant higher-order oligomerization states as appear when the slowest dynamic mode is excluded.

Supplementary Tables:

Supplementary Table 1: Crystallographic Summary of MA_IP6 crystal structures

	MA_IP6_R32	MA_IP6_C2	MA_IP6_SFX_P1
Data Collection			
Space group	*R*32	*C*2	*P*1
Cell Dimensions			
a, *b*, *c* (Å)	97.83, 97.83, 176.30	176.18, 67.41, 97.50	96.60, 96.60, 91.10
α, β, γ (°)	90.00, 90.00, 120.00	90.00, 123.15, 90.00	90.00, 90.00, 120.00
Resolution* (Å)	39.10 - 2.40 (2.64 - 2.40)	44.41 - 2.72 (2.82 - 2.72)	24.57 - 3.30 (3.45 - 3.30)
R_{merge}	0.214 (1.54)	0.0988 (0.799)	
XFEL R_{split}			0.315 (6.139)
I/σI	13.21 (2.20)	15.74 (2.42)	2.67 (0.18)
Completeness	99.55 (99.53)	99.82 (99.88)	100.0 (100.0)
Redundancy	20.3 (21.2)	6.8 (7.0)	62.3 (36.1)
CC1/2	0.998 (0.858)	0.998 (0.845)	0.958 (0.087)
Refinement			
Resolution (Å)	39.10 - 2.40 (2.64 - 2.40)	44.41 - 2.72 (2.82 - 2.72)	24.57 - 3.30 (3.45 - 3.30)
No. of reflections	12,931 (3,027)	25,957 (2,732)	35,663 (2,987)
R_{work}	0.220	0.213	0.351
R_{free}	0.276	0.272	0.408
No. of Atoms			
Protein	1,920	5,349	11,415
Ligand	126	324	504
Water	97	69	80
B factors			

Protein	48.06	65.11	174.54
Ligand	114.18	140.07	81.75
Waters	57.26	54.86	56.99
RMSD			
Bond length (Å)	0.018	0.022	0.001
Bond angles (°)	1.99	1.96	0.464
PDB ID	7E1K	7E1J	7E1I

Supplementary Table 2: Root-mean-square deviation (RMSD) among MA structures

		RMSD (Å) of Cα atoms of chain A†		
	Coordinate error (Å)*	MA_IP6_R32	1HIW	2HMX
MA_IP6_C2	0.37	0.340	0.279	1.32
MA_IP6_R32	0.28	-	0.273	1.38
MA_IP6_SFX_P1	0.78	0.580	0.553	1.48
1HIW	-	-	-	1.34

Supplementary Table 3: Root-mean-square deviation (RMSD) among MA structures

	RMSD (Å) of Cα atoms of chain A†			
	2H3F	2H3I	2H3Q	2H3Z
MA_IP6_C2	0.864	0.737	0.742	0.818

Supplementary Table 4: Average *B*-factor and contact area with MA of IP6 molecules

Objects	*B*-factor ($Å^2$)	Contact area ($Å^2$)
MA	65.08	
IP6_1	182.97	214.2
IP6_2	120.05	161.1
IP6_3	144.01	136.2
IP6_4	161.55	155.9
IP6_5	129.75	
IP6_6	117.63	

Supplementary Figures

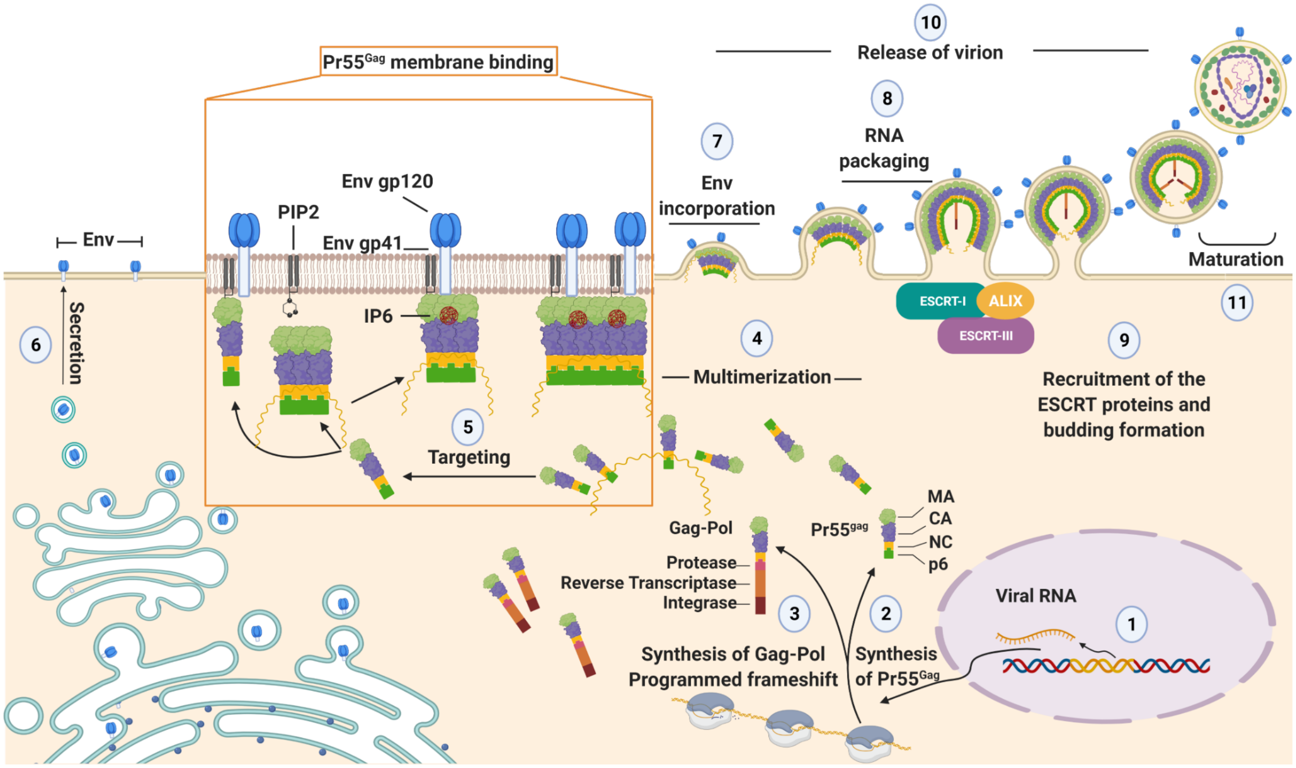

Supplementary Fig. 1: Representation of the late-stage of the HIV-1 cycle. The viral RNA is transcribed and exported from the nucleus to the cytoplasm (1). It acts as a template for the translation of Pr55^{Gag} precursor polyproteins (2). The Gag-Pol polyprotein synthesis requires a programmed frame-shifting process during translation (3). Pr55^{Gag} initiates multimerization by recruiting viral genomic RNA (4) and targets the plasma membrane for assembly through its MA domain (5). After multimerized Pr55^{Gag} polyproteins are anchored to the membrane via their amino-terminal myristate, they incorporate with Env (7), which arrives at the plasma membrane via a secretory pathway from the Golgi and RER (6). During the RNA packaging (8) and budding process, ESCRT-I and ESCRT-III are recruited to perform a membrane scission (9) which is characterized by the release of the virion particle (10). Finally, maturation (11) is catalyzed by proteolytic cleavage of the viral protease for canonical capsid core formation. **ALIX**: (ESCRT-associated factor) ALG2-interacting protein X, **CA**: capsid domain, **Env**: viral envelope glycoprotein, **ESCRT-I**: endosomal sorting complex required for transport I, **MA**: matrix domain, **NC**: nucleocapsid domain.

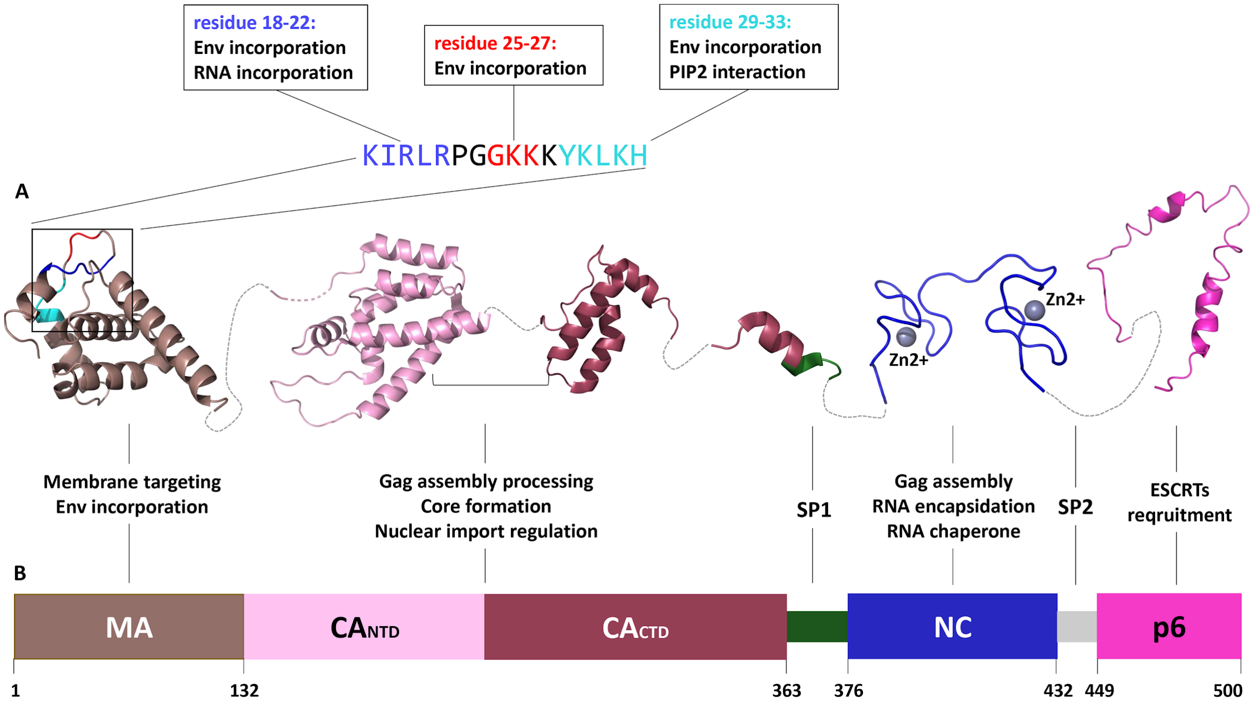

Supplementary Fig. 2: Representation of the Gag precursor polyprotein domains. a Structural model of the separated and extended Gag domains. The significance of the N-terminal of the MA domain is shown sequentially, and its description is demonstrated in the boxes. The domains are assembled by high-resolution structures available in Protein Data Bank (PDB). Linker regions are shown with dashed lines. **b** Schematic representation of the putative Gag domain boundaries. The beginning and end of each domain are labeled as the residue number and the functions of corresponding domains are indicated.

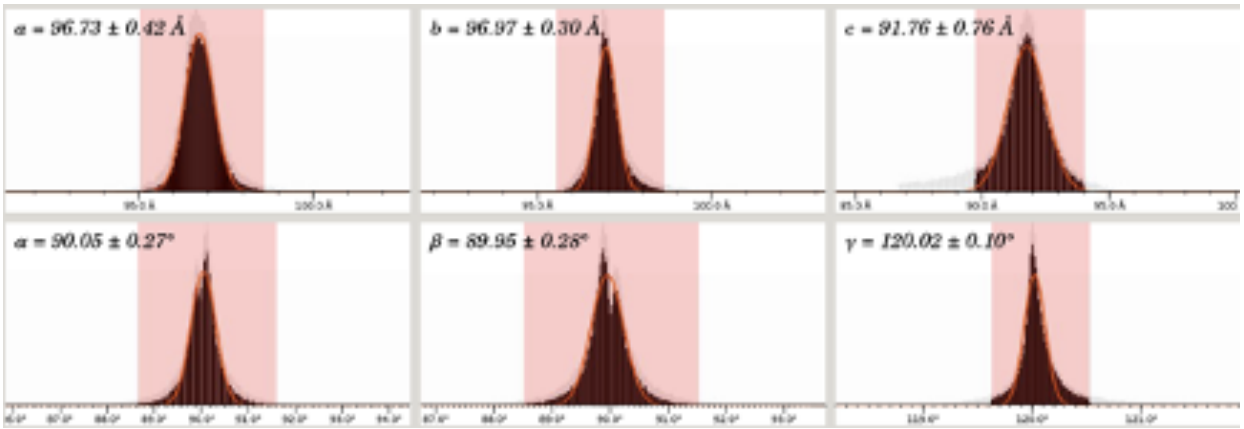

Supplementary Fig. 3: Unit cells of MA_IP6_SFX dataset. Indexing cell file generated by *CrystFEL* unit cell file version 1.0

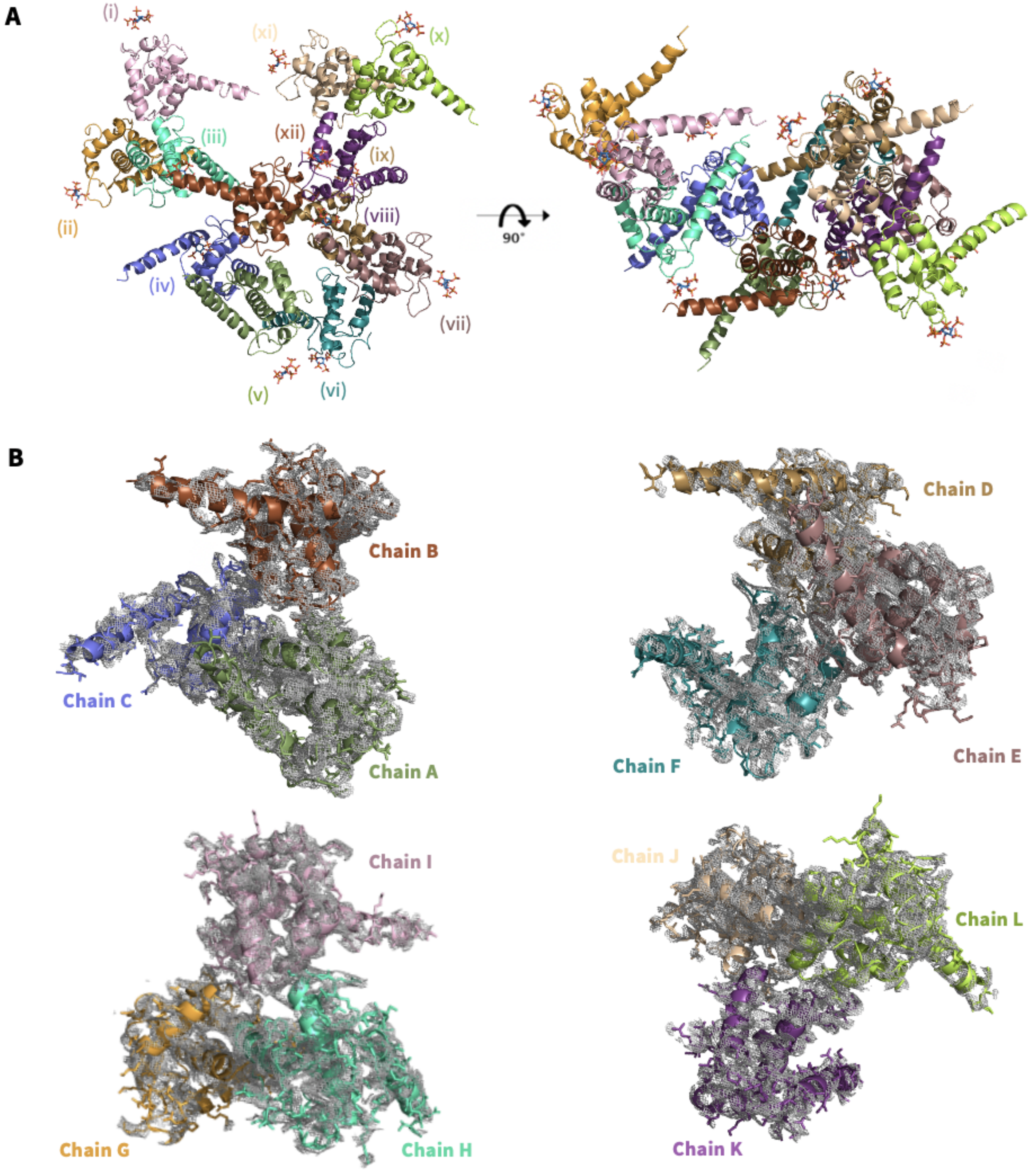

Supplementary Fig. 4: SFX structure of MA_IP6_SFX_P1 complex. a The overall structure of the MA_IP6_SFX_P1 complex is colored based on each chain. There are a total of 16 molecules in the asymmetric unit cell. Carbon, oxygen and phosphorus atoms of IP6 molecules are colored by sky-blue, red and orange, respectively. **b** A 2*F*o-*F*c simulated annealing-omit map for each trimer is shown in the gray mesh.

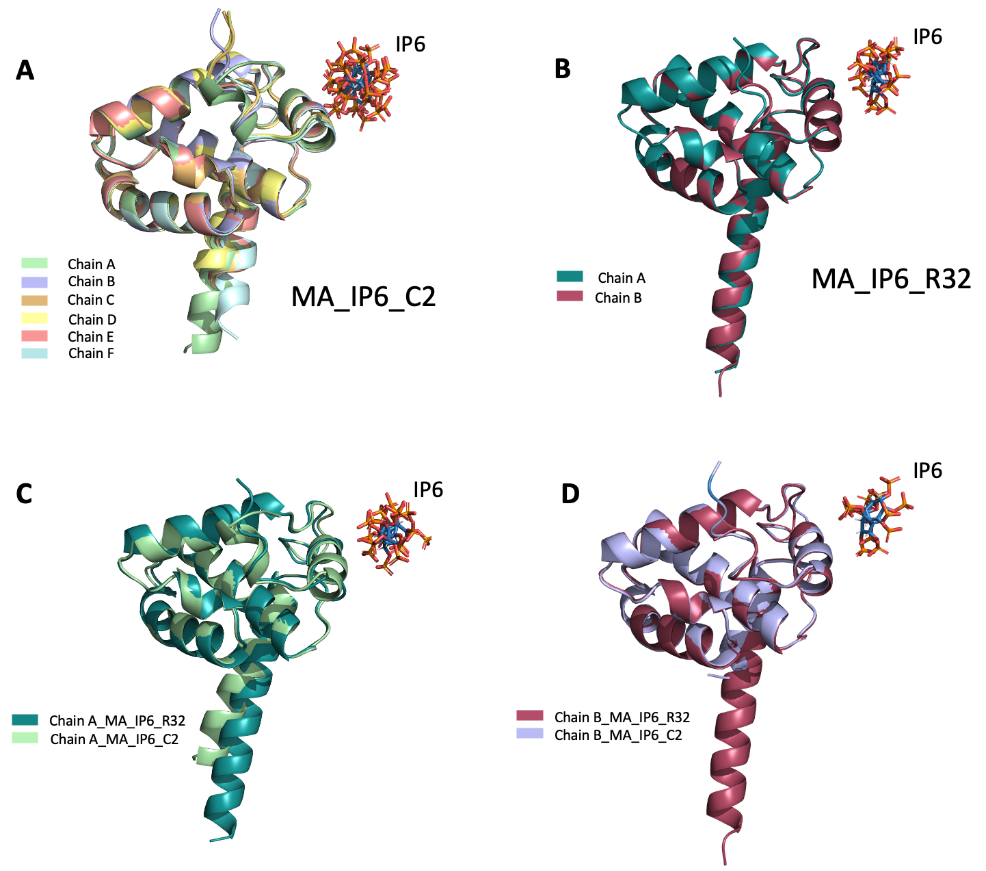

Supplementary Fig. 5: Superposition of chains for two crystal forms. a Each chain of MA_IP6_C2 complex is superposed with an overall RMSD of 0.286 Å. **b** Two chains of the MA_IP6_R32 complex are superposed with an RMSD of 0.200 Å. **c** Chain A of MA_IP_C2 complex is superposed with chain A of MA_IP6_R32 complex with RMSD of 0.370 Å. **d** Chain B of MA_IP_C2 complex is superposed with chain B of MA_IP6_R32 complex with RMSD of 0.317 Å.

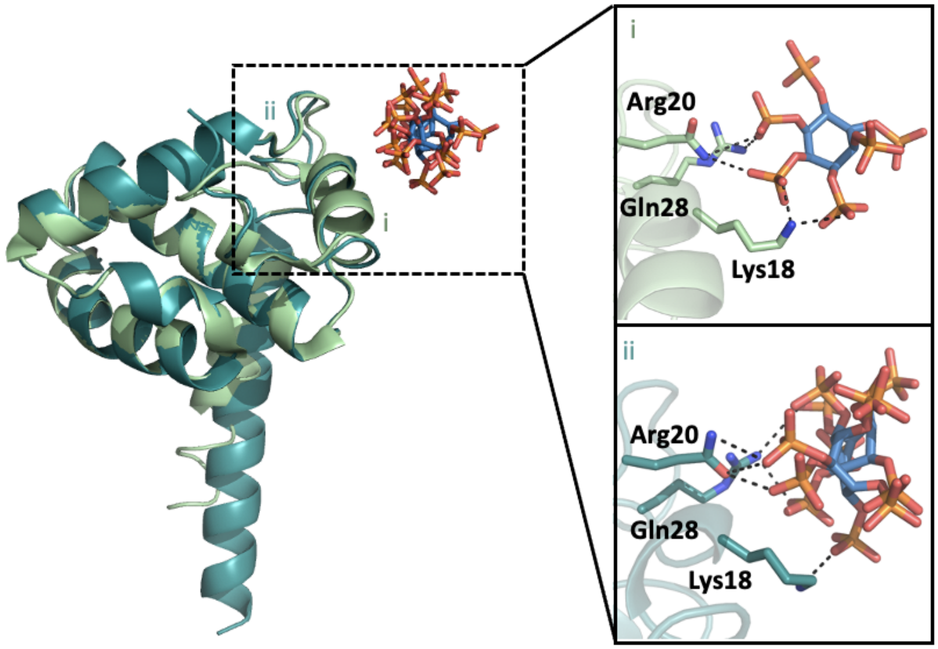

Supplementary Fig. 6: Comparison of active residues in the binding pocket for the chain A of MA_IP6_C2 (i) and MA_IP6_R32 (ii) structures. Chain A of MA_IP_C2 (Pale green) complex is superposed with chain A of MA_IP6_R32 (Deep teal) complex with RMSD of 0.370 Å. Hydrogen bonds are shown with dashed lines.

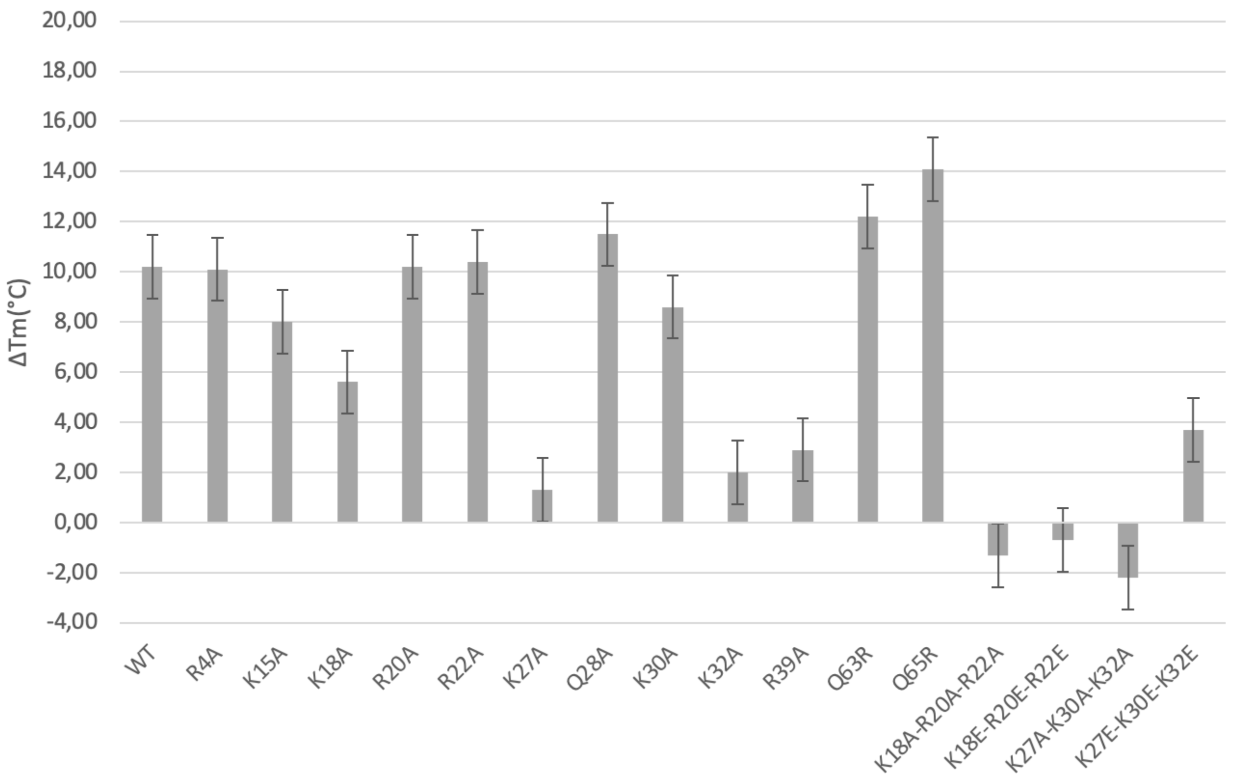

Supplementary Fig. 7: DSF assay results of point mutations of MA. The change of melting temperature (ΔTm) in the presence and absence of IP6, respectively for the wild-type and mutant MA proteins are indicated with grey bars.

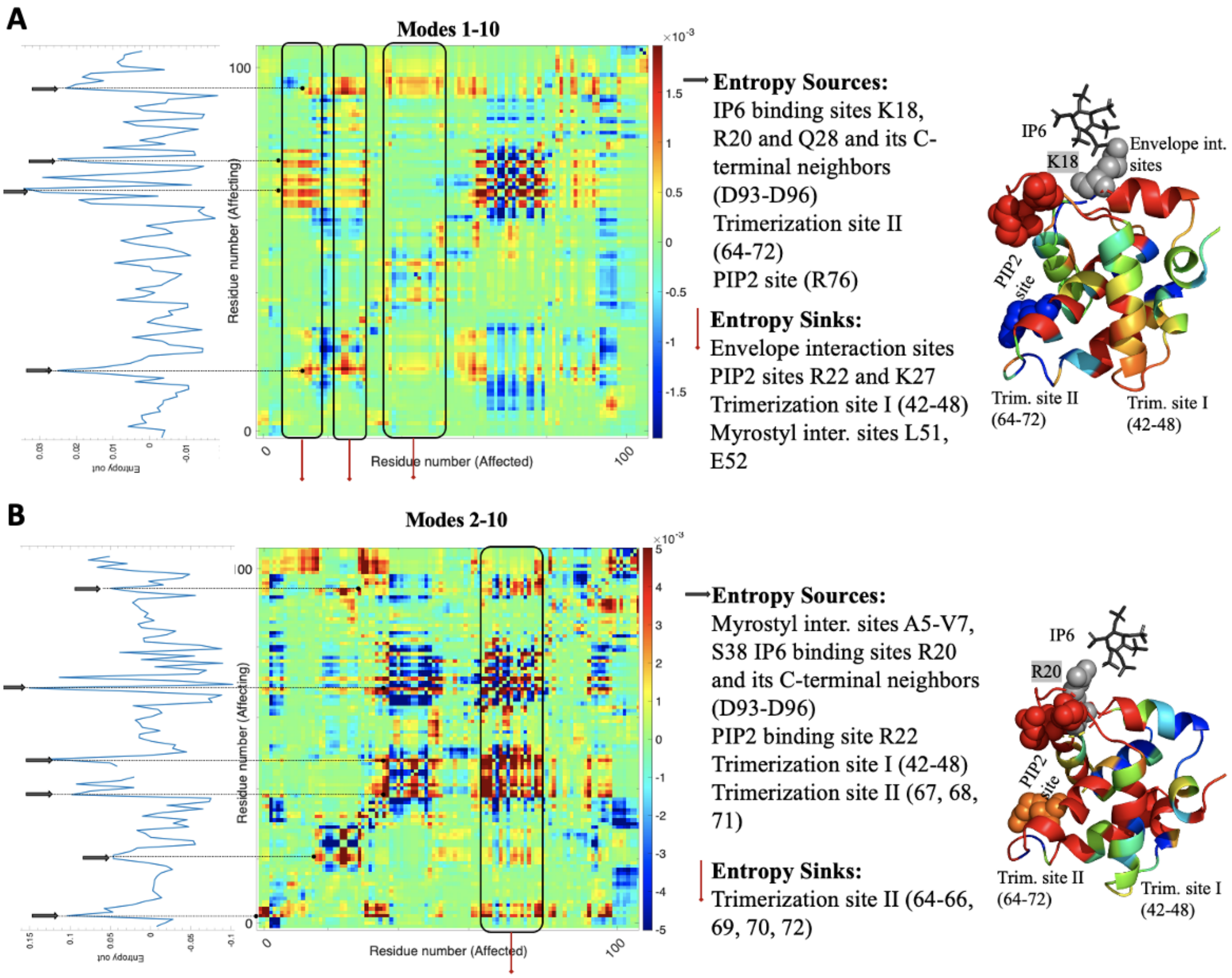

Supplementary Fig. 8: Net transfer entropy (nTE) maps of the monomer (MA_IP6_C2) showing the causal interrelation for each residue pair (affecting versus affected) in averages of first ten (A) and two to ten (B) slowest modes. Cumulative net transfer entropy (cnTE) plots extracted from each nTE map are given on the left. As exemplary cases, the causal interrelations of residues K18 **(A)** and R20 **(B)** (gray spheres) with the rest of residues are color-coded on the monomer, from highest positive (red) to lowest negative (blue) nTE values. Entropy sources and entropy sinks for chain A are listed and indicated respectively with black and red arrows.

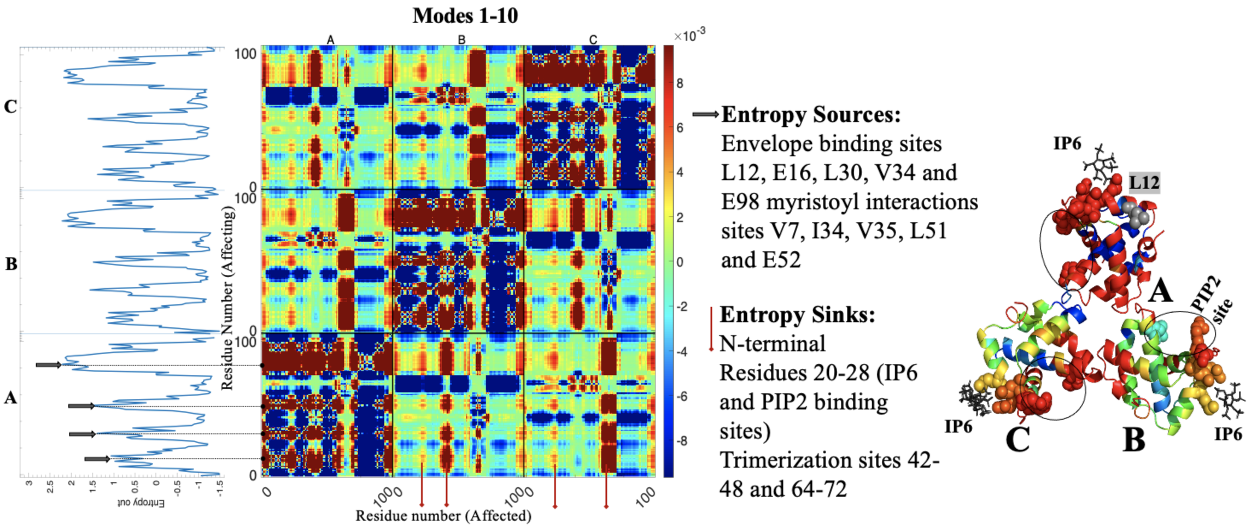

Supplementary Fig. 9: Net transfer entropy (nTE) map of the trimer (MA_IP6_C2) showing the causal interrelation for each residue pair (affecting versus affected) in average of first ten slowest modes. Cumulative net transfer entropy (cnTE) plot extracted from the nTE map is given on the left. As an exemplary case, the causal interrelation of L12 (gray spheres, chain A) with the rest of residues are shown on the trimer, color-coded from highest positive (red) to lowest negative (blue) nTE values. Entropy sources and entropy sinks are listed and indicated respectively with black (chain A) and red (chains B and C) arrows.

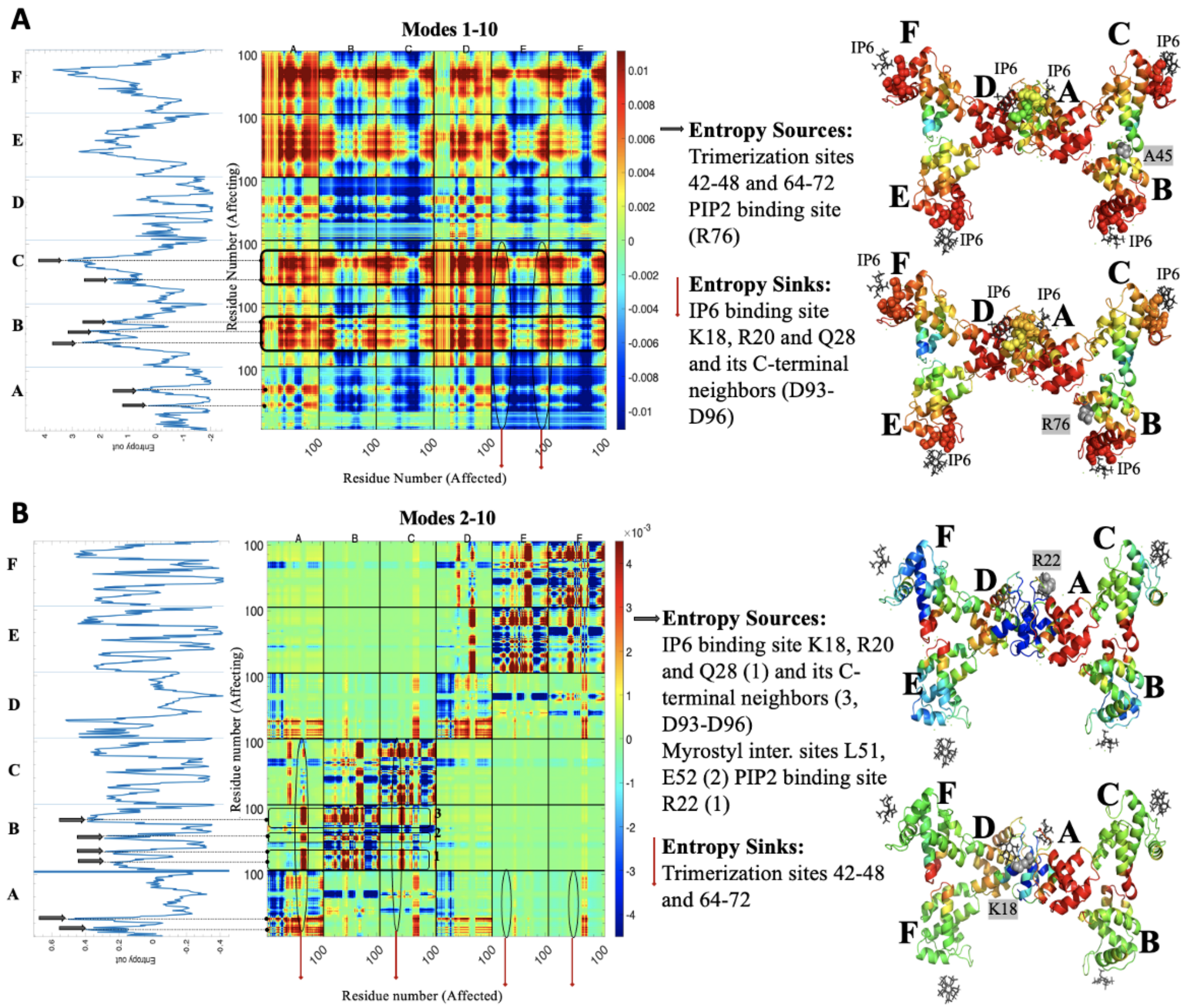

Supplementary Fig. 10: Net transfer entropy (nTE) map of the hexamer (MA_IP6_C2) showing the causal interrelation for each residue pair (affecting versus affected) in averages of first ten (A) and two to ten (B) slowest modes. Cumulative net transfer entropy (cnTE) plots extracted from each nTE map are given on the left. As exemplary cases, the causal interrelation of residues A45 and R76 on chain B **(A)** and K18 and R22 on chain A **(B)** (gray spheres) with the rest of residues are color-coded on the hexamer, from highest positive (red) to lowest negative (blue) nTE values. Entropy sources (on chains A, B, C **(A)** and chains A, B **(B)**) and entropy sinks (on chain E **(A)** and chains A, C, E, F **(B)**) are listed and indicated respectively with black and red arrows.

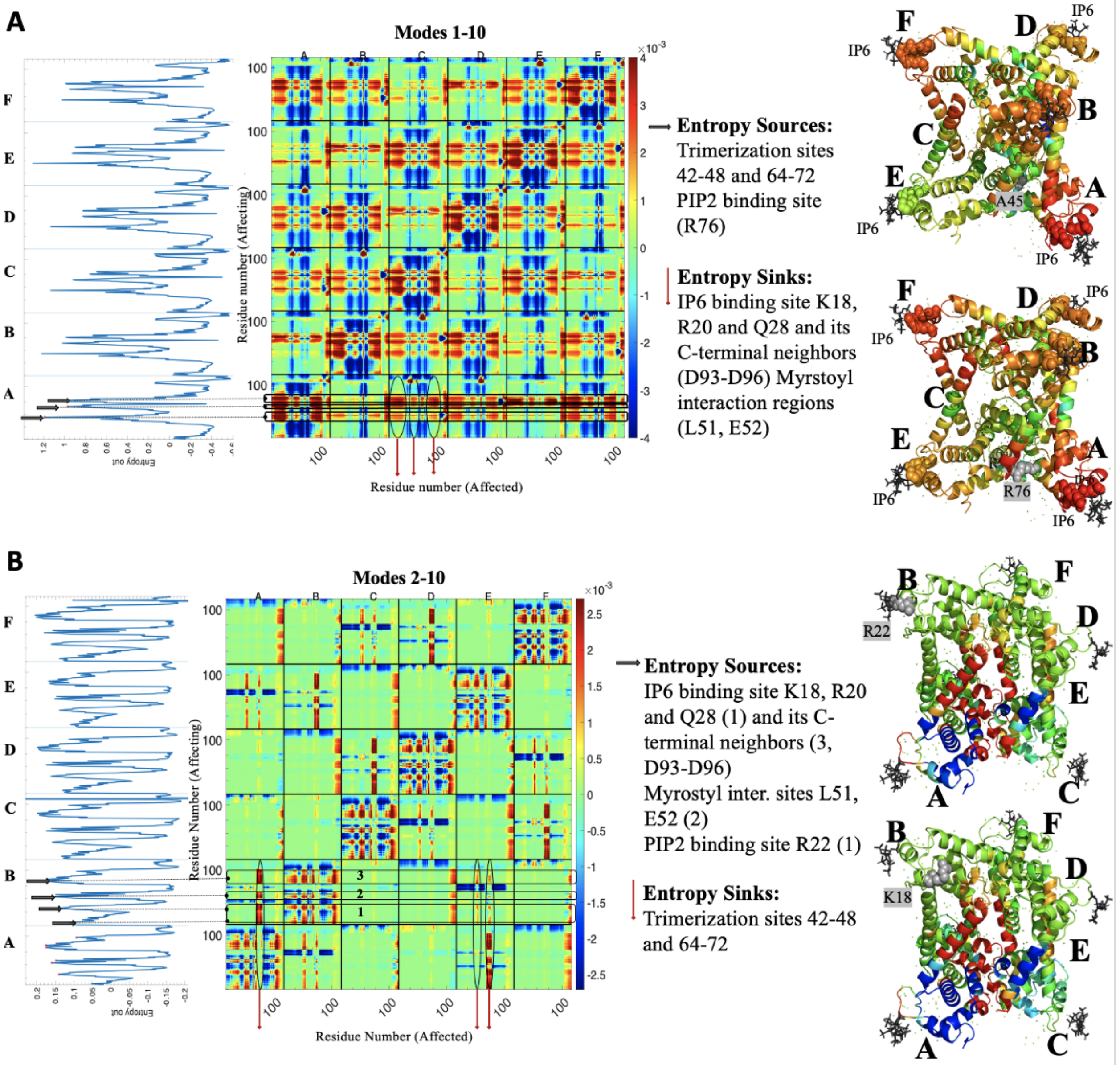

Supplementary Fig. 11: Net transfer entropy (nTE) map of the hexamer (MA_IP6_R32) showing the causal interrelation for each residue pair (affecting versus affected) in averages of first ten (A) and two to ten (B) slowest modes. Cumulative net transfer entropy (cnTE) plots extracted from each nTE map are given on the left. As exemplary cases, the causal interrelation of A45 and R76 on chain A **(A)** and K18 and R22 on chain B **(B)** (gray spheres) with the rest of residues is color-coded on the hexamer, from highest positive (red) to lowest negative (blue) nTE values. Entropy sources for chain A **(A)** and chain B (shown in rectangles as 1-IP6 and PIP2 binding sites, 2-myristoyl interaction sites and 3-C-terminal IP6 neighbors on **B**) and entropy sinks for chain C **(A)** and chains A and E **(B)** are listed and indicated respectively with black and red arrows.

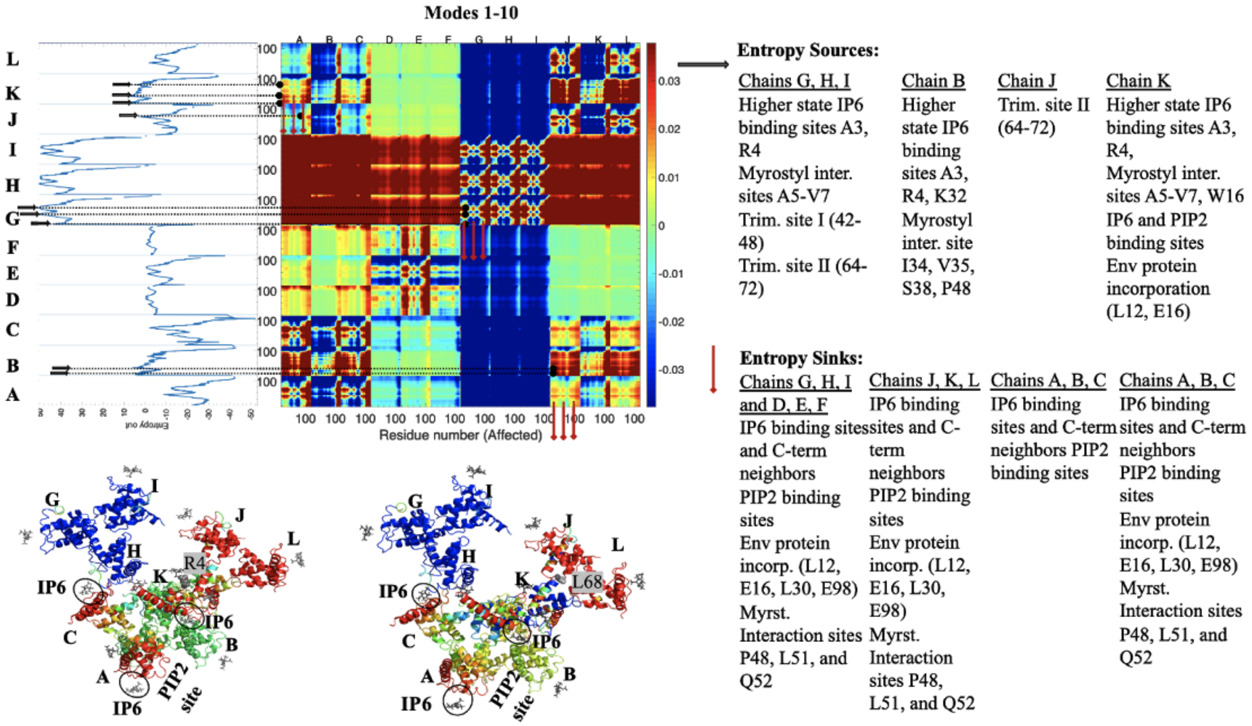

Supplementary Fig. 12: Net transfer entropy (nTE) map of the dodecamer (MA_IP6_P1) showing the causal interrelation for each residue pair (affecting versus affected) in average ten slowest modes. Cumulative net transfer entropy (cnTE) plot extracted from the nTE map is given on the left. Below the nTE map, as exemplary cases, the causal interrelations of R4 (chain K) and L68 (chain J) (gray spheres) with the rest of the residues on the dodecamer, color-coded from the highest positive (red) to lowest negative (blue) nTE values. Entropy sources on chains B, G, J and K and entropy sinks on chain J are listed and indicated respectively with black and red arrows.

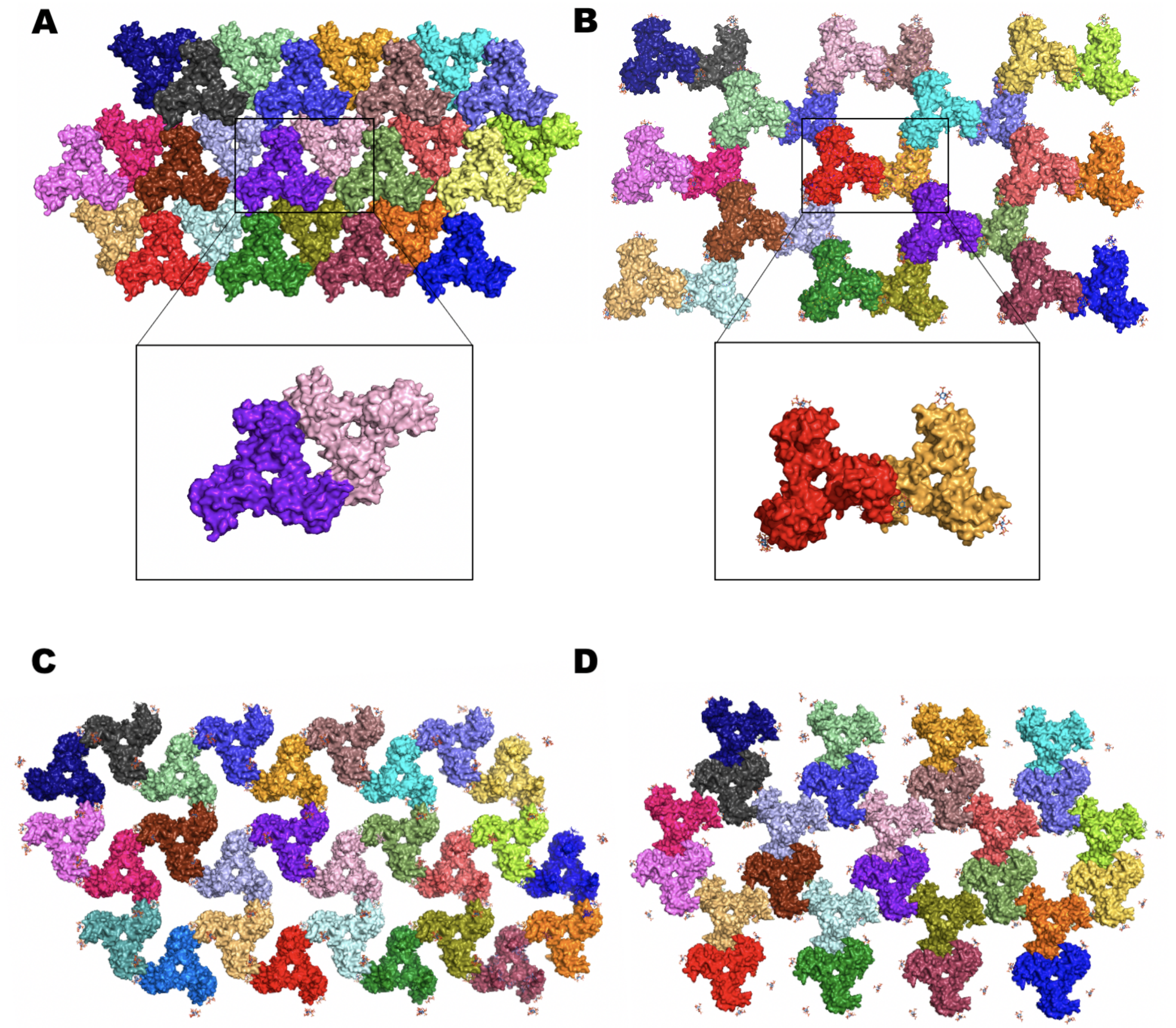

Supplementary Fig. 13: Comparison of positions of trimers in three HIV-MA structures. Three different positions of trimer are shown in 1HIW, MA_IP6_C2, MA_IP6_R32 and MA_SFX structures, respectively. **a** Estimated assembly of 1HIW structure without IP6. The asymmetric unit is shown with the purple-blue and light pink with surface representation. **b** Assembly of MA_IP6_C2 in the presence of IP6. The asymmetric units are shown with red and bright orange surfaces, respectively. **c** Trimer assembly of MA_IP6_R32 structure **d** Trimer assembly of MA_SFX structure.

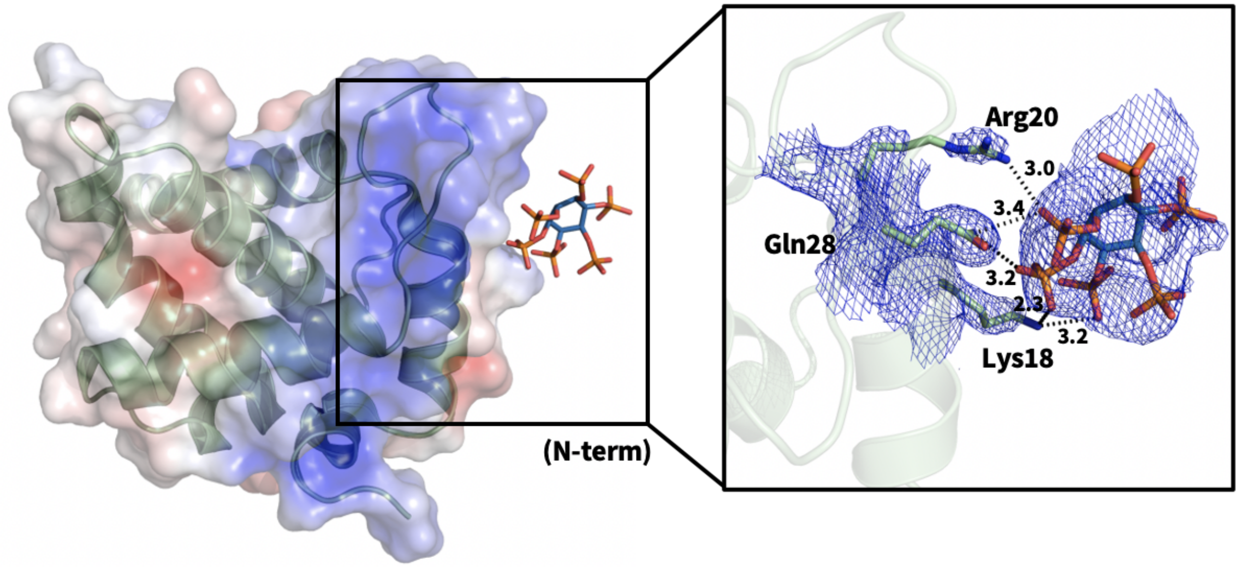

Supplementary Fig. 14: HBR within N- terminus of MA and interaction with IP6. Chain A of MA_IP6_C2 structure is colored in pale-green and carbon, oxygen and phosphorus atoms of IP6 are colored by sky-blue, red and orange, respectively with their electron density map. Polar contacts are shown with dotted lines in Angstrom. The surface of the MA_IP6_C2 structure is represented by using APBS electrostatic. Basic regions are represented by blue, acidic regions by red and hydrophobic surfaces are colored in gray.

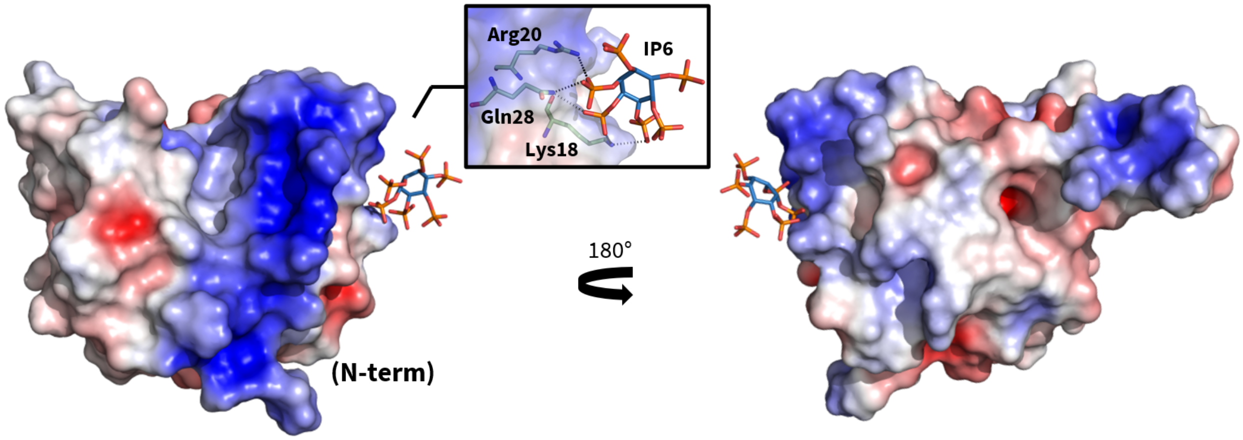

Supplementary Fig. 15: Representation of electrostatic potential surface for chain A of MA_IP6_C2 structure. APBS electrostatic is used to detect the highly basic region within the N-terminal via *PyMOL* and the structure is rotated 180 degrees in the y-axis.
